# The Role of Temperature in Moral Decision-Making: Limited Reproducibility

**DOI:** 10.3389/fpsyg.2021.681527

**Published:** 2021-09-28

**Authors:** Ryunosuke Sudo, Satoshi F. Nakashima, Masatoshi Ukezono, Yuji Takano, Johan Lauwereyns

**Affiliations:** ^1^Graduate School of Systems Life Sciences, Kyushu University, Fukuoka, Japan; ^2^Department of Education and Psychology, Kagoshima Immaculate Heart University, Satsuma-Sendai-Shi, Japan; ^3^Department of Developmental Disorders, National Center of Neurology and Psychiatry, National Institute of Mental Health, Kodaira, Japan; ^4^Department of Psychology, University of Human Environments, Okazaki, Japan; ^5^School of Interdisciplinary Science and Innovation, Kyushu University, Fukuoka, Japan; ^6^Faculty of Arts and Science, Kyushu University, Fukuoka, Japan

**Keywords:** moral dilemma, temperature, cold, environment, reproducibility

## Abstract

Temperature is one of the major environmental factors that people are exposed to on a daily basis, often in conditions that do not afford control. It is known that heat and cold can influence a person’s productivity and performance in simple tasks. With respect to social cognition, it has also been suggested that temperature impacts on relatively high-level forms of decision-making. For instance, previous research demonstrated that cold temperature promotes utilitarian judgment in a moral dilemma task. This effect could be due to psychological processing, when a cool temperature primes a set of internal representations (associated with “coldness”). Alternatively, the promotion of utilitarian judgment in cold conditions could be due to physiological interference from temperature, impeding on social cognition. Refuting both explanations of psychological or physiological processing, however, it has been suggested that there may be problems of reproducibility in the literature on temperature modulating complex or abstract information processing. To examine the role of temperature in moral decision-making, we conducted a series of experiments using ambient and haptic temperature with careful manipulation checks and modified task methodology. Experiment 1 manipulated room temperature with cool (21°C), control (24°C) and hot (27°C) conditions and found only a cool temperature effect, promoting utilitarian judgment as in the previous study. Experiment 2 manipulated the intensity of haptic temperature but failed to obtain the cool temperature effect. Experiments 3 and 4 examined the generalizability of the cool ambient temperature effect with another moral judgment task and with manipulation of exposure duration. However, again there were no cool temperature effects, suggesting a lack of reproducibility. Despite successful manipulations of temperature in all four experiments, as measured in body temperature and the participants’ self-reported perception, we found no systematic influence of temperature on moral decision-making. A Bayesian meta-analysis of the four experiments showed that the overall data tended to provide strong support in favor of the null hypothesis. We propose that, at least in the range of temperatures from 21 to 27°C, the cool temperature effect in moral decision-making is not a robust phenomenon.

## Introduction

Human cognition, judgment, and behavior are usually studied in psychology in the relatively controlled context of a laboratory. However, the environment is an important factor which influences cognition and decision-making. Temperature is one of the major environmental factors, and people are constantly exposed to it. As for the effects of temperature on behavior, for example, climate change including temperature is considered to be one of the principal drivers of human migration from the macroscopic perspective of society and group aggregate (Büntgen et al., 2011). Temperature can reduce the economic productivity of societies (Burke et al., 2015b) and increase the frequency of conflict and violent crime (Zhang et al., 2007; Ranson, 2014; Burke et al., 2015a).

From the microscopic perspective of the individual, it has been demonstrated that temperature affects human cognitive function and performance of simple tasks (Pilcher et al., 2002; Hancock et al., 2007; Yeganeh et al., 2018). In particular, these studies showed that cognitive abilities and productivity were consistently reduced in high intensity hot and cold environments. It was also indicated that temperature affects mood and emotion (Anderson, 2001; Keller et al., 2005). From these previous studies, it seems plausible that temperature can affect human emotions, cognitive function, and work performance. However, to what extent can it affect social cognition?

Studies of decision-making, particularly social judgment, reported that a warm temperature tends to make legal judgments stricter (Heyes and Saberian, 2019) and increases the frequency of dead-ball rulings in baseball games (Larrick et al., 2011). It has also been reported that a hot temperature promotes prosocial behavior (Williams and Bargh, 2008) and enhances cooperative behavior in prisoner’s dilemma tasks (Storey and Workman, 2013). On the other hand, research has shown that a cold temperature induces lower investment in trust games (Kang et al., 2011), tougher inferences of suspect guilt (Gockel et al., 2014), and more utilitarian judgments in moral dilemma tasks (Nakamura et al., 2014). These studies suggested that temperature has a considerable impact on real-world behavior beyond simple task performance. Especially, the fact that cooperative behavior and moral judgments can be affected is an important finding when considering the behavior in uncontrollable temperature environments.

At the most general theoretical level, there may be two divergent types of explanations for temperature effects on social cognition. On the one hand, the physical temperature could affect people psychologically, by activating internal representations associated with different temperatures. For instance, Williams and Bargh (2008) interpreted their observations as a form of social priming, called temperature priming, whose mechanism is the activation of specific concepts linked to temperature based on embodied cognition (see also IJzerman and Semin, 2009; Gockel et al., 2014; Steinmetz and Posten, 2017; Wang, 2017). Specifically, the physiological experience of warm temperature connects to an interpersonal impression of warmth; in terms of the perception of psychological distance, a warm temperature induces an impression of close social proximity. Cool temperature induces the opposite effects.

On the other hand, the effect from physical temperature on cognition could have a more physiological basis, in terms of either in terms of heat stress or cold stress, where the extent of the impact on executive brain functions depends on the intensity of the stressor (e.g., Taylor et al., 2016; Abbasi et al., 2019).

Before aiming to tease apart psychological vs. physiological mechanisms, however, we should confirm the most basic facts of any phenomena. Researchers have raised the problem of the reproducibility of findings with respect to the effect of temperature on social cognition (e.g., Doyen et al., 2012). Previous experimental studies typically did not apply preregistration procedures (except the replication study by Lynott et al., 2014); a few studies conducted sample size calculation and power analysis (Gockel et al., 2014; Nakamura et al., 2014; Steinmetz and Posten, 2017).

Although studies after Williams and Bargh (2008) could be considered as a kind of conceptual replication of their study, the quality of the evidence is questionable in terms of reproducibility due to procedure problems when measuring temperature effects. In fact, Lynott et al. (2014, 2017) and Chabris et al. (2019) conducted direct replication studies of the pioneering temperature priming work by Williams and Bargh (2008) but failed to obtain similar findings. LeBel and Campbell (2013) and Pashler et al. (2012) also reported failures to replicate other temperature priming studies. On the other hand, there were reports of successful replication. Schilder et al. (2015) conducted a direct replication study of IJzerman and Semin (2009) and obtained similar results. Bargh and Melninkoff (2019) reanalyzed the data from a study that showed a failed replication (Chabris et al., 2019) and found a trend similar to that of the original study. As such, the question of reproducibility remains wide open.

The notion that social judgment can be impacted by uncontrollable temperature environment has important implications for our understanding of real-life decision-making under a variety of stressors; the issue of reproducibility, therefore, deserves to be inspected carefully. In the present study, we aimed to reexamine the role of temperature in moral decision-making by extending the paradigm employed by Nakamura et al. (2014), conducting a series of experiments using ambient and haptic temperature with careful manipulation checks and modified task methodology.

## Experiment 1

Nakamura et al. (2014) investigated the effect of haptic temperature on moral judgments using the moral dilemma task. In this task, the offered dilemmas reflect situations in which the subject can save a larger number of lives by sacrificing one person’s life. Nakamura et al. (2014) showed that cool temperature enhances utilitarian judgment in the sense that subjects tended to opt for saving more people in the moral dilemma task. The cool temperature effect was induced by having the subjects wear a scarf with frozen water packs as opposed to a scarf with water packs at room temperature.

Here, in Experiment 1, we adapted the paradigm to investigate the effect of ambient environmental temperature. The experimental conditions and task and procedures were changed from Nakamura et al. (2014) as follows.

While the original study compared two conditions (Cold vs. Control), we examined three conditions, adding a hot temperature condition. In many temperature studies on priming, the direction of the effect of hot and cold temperatures is opposite. We investigated whether the same tendency is observed in moral judgment. The three conditions were controlled at room temperature to examine the effect of ambient temperature. 21°C was cool temperature condition and 24°C was control temperature condition, and 27°C was hot temperature condition. The temperature settings were determined within a range where people feel no discomfort (between 17°C and 28°C) as defined by the Japanese Industrial Safety and Health Law, taking into account the ethical guidelines at the university where the experiment was conducted. Specifically, Cool (21°C) and Hot (27°C) were set as the experimental temperature conditions which could be stably controlled, and 24°C, the middle value between the two experimental conditions, was adopted as the control.

Next, we updated the moral judgment task used in the experiment to the latest version. The original study used the moral dilemma task by Greene et al. (2001), which has been criticized with respect to its content validity. Christensen et al. (2014) developed a revised moral dilemma task which carefully controls the design of each item in terms of word count and description style and systematically composed the items with respect to conceptual factors (Personal Force, Benefit Recipient, Evitability, and Intention). We used the new version of the task by Christensen et al. (2014) in this experiment to carefully control the composition of the dilemmas.

Nakamura et al. (2014), when manipulating temperature using a scarf, used deception to suggest that the temperature manipulation and moral judgment were completely different experiments. Here, we did not apply such deception since the temperature manipulation was ambient temperature, not tactile temperature through an object. We employed a manipulation check on the experimental temperature condition to measure the validity of the objective temperature manipulation, independent from the task performance. In addition, in accordance with the university’s ethical guidelines, the participants were informed at the start of the experiment that this study related to temperature. However, the participants were not informed about the experimental conditions and research hypotheses. Thus, the details of the experimental conditions were unknown to the participants, such as how many conditions there were in this study and whether there were hotter or colder conditions than their own. Accordingly, we assumed that any specific effect from temperature would derive from implicit processes in this study, comparable to other temperature studies.

Finally, Nakamura et al. (2014) used questionnaires to assess different aspects of mindset and affect. They used these questionnaires in an effort to tease apart two theoretical alternatives about the mechanisms underlying the temperature effect on decision-making in the moral dilemma task (one based on mindset, one based on affect). Here, given time constraints in the experimental sessions, we opted not to use these questionnaires. Our basic aim was to establish a straightforward relationship between ambient temperature and moral judgment, without as yet engaging in a debate on the underlying mechanisms.

In line with the findings by Nakamura et al. (2014), it was expected that cool temperature promotes utilitarian judgment; in contrast, hot temperature would inhibit utilitarian judgment.

### Method

#### Participants

The priori sample size calculation using G*power (Faul et al., 2007) was conducted based on the results of Experiment 1 in Nakamura et al. (2014) with power of 0.80, and alpha of 0.05. When assuming to observe an interaction between temperature and dilemma scenario as in the previous study, the calculation with effect size *f*=0.295 indicated a minimum sample size of 11 per group. To detect a main effect of temperature, the calculation with effect size *f*=0.288 indicated a minimum sample size of 22 per group. Considering the possibility that ambient temperature has a different effect from haptic temperature, we focused on the main effect observation and decided to collect at least 22 participants per experimental condition.

Participants were 82 healthy Japanese undergraduate and graduate students (39 males and 43 females, age: *M*_age_=20.15, *SD_age_*=1.81). Participants were randomly assigned to one of the three different room temperature conditions: 24°C as Control (14 males, 13 females), 21°C as Cool (12 males, 16 females), and 27°C as Hot (13 males, 14 females). The number of participants in each condition was unbalanced due to unexpected cancellation. The present study was conducted during two seasons to control for the seasonal effect: July and August as summer (24 males, 14 females) in Tokyo and January and February as winter (15 males, 29 females) in Hiroshima. All subjects were naïve to the purposes of the experiment. Each subject received compensation worth 1,500 yen for their participation. Written informed consent was obtained from all participants before the experiment.

#### Apparatus

A laptop computer with Psychopy software (version 1.85.1; Peirce et al., 2019) controlled all events and data collection. The computer was connected to an independent keyboard and a 24.5-inch monitor with a display resolution of 1920×1080 pixels on the desk, and the computer itself was hidden from participants.

### Measures

#### Physical Measurements

The room temperature was monitored with a temperature measurement device (THD501, Citizen Systems Japan Co., Ltd., Japan), and it was recorded approximately every 15min during the experiment.

#### Physiological Measurements

The skin surface temperature of the forehead was measured with non-contact infrared thermometers (DM300, AEDON, LLC., Russia).

#### Subjective Measurements

The questionnaire used to obtain subjective feelings included questions regarding room warmth, comfort and arousal by 7-point scale. The participants were asked, first, to rate their perceived level of room warmth (1 = very cool; 7 = very warm), second, to indicate their level of comfort (1 = very uncomfortable; 7 = very comfortable) and, third, to indicate their level of arousal (1 = very calm; 7 = very excited). All scenarios in the dilemma task were controlled so as to achieve the greater good by sacrificial harm.

#### Moral Judgment Task

Sixteen scenarios were selected from a battery of 46 moral dilemmas developed by Christensen et al. (2014) and they were presented in random order. They were translated into Japanese and checked by an English-Japanese bilingual speaker. The scenario IDs used in the present study were 7, 8, 11, 12, 13, 14, 19, 20, 25, 26, 33, 34, 37, 38, 47, and 48 in the original study (Christensen et al., 2014). There were 4 scenario factors (Personal force, Evitability, Benefit receptor, and Intentionality) that could affect judgment in moral dilemmas. To create a dilemma set composed of two items per combination of scenario factors, the factor of Intentionality was fixed to only Instrumental, and 16 items were selected by factorially combining the other three factors. Participants were asked to rate the moral acceptability of the proposed utilitarian action in each dilemma using a 7-point scale (1 = Completely unacceptable; 7 = Completely acceptable).

### Procedure

Participants were tested individually. At the beginning of the experiment, the experimental procedure was explained to them in a waiting room for about 10min. This period also served to control their condition before the temperature manipulation. Informed consent was obtained from each participant during this period. The waiting room temperature was set at 24°C (*M_temperature_*=22.31, *SD_temperature_*=2.87), the same temperature as the control condition, through the room air conditioner.

After the instruction, the skin temperature of the participants was measured as the baseline (Time 1) of their physiological level. Then, the participants entered the experiment room where the temperature was controlled as according to each experimental condition, Cool (*M_temperature_*=21.02, *SD_temperature_*=0.44), Control (*M_temperature_*=24.15, *SD_temperature_*=0.39), and Hot (*M_temperature_*=27.41, *SD_temperature_*=0.44) by setting the room air conditioner with circulator and heater. Since the experiment room was adjacent to the waiting room, the participants could move to the experiment room directly from the waiting room without passing through any other rooms. First, the participants waited for 10min alone in the experiment room while relaxing to habituate to the environment. After the habituation period, they performed three cognitive tasks unrelated to the moral dilemma task (facial expression judgment, face recognition, calculation) for about 60min with the temperature as according to the experimental condition. Each task took about 15min. At the beginning of the set of tasks, including the moral dilemma task, the participants were given instructions by the experimenter about the task procedures with a practice session and then performed the tasks alone in the room after the instruction. After they had completed the three unrelated cognitive tasks, the participants filled out the questionnaire about the subjective room warmth and their feelings of comfort and arousal; also, their skin temperature was measured before the moral dilemma task as a manipulation check (Time 2).

The participants were asked to perform the moral dilemma task after the manipulation check. The procedure of the moral dilemma task was based on Christensen et al. (2014). Each dilemma was presented as text through a sequence of three screens. The first screen contained the first paragraph of the scenario and was presented upon pressing the spacebar. With the next keypress, the second screen appeared with a new paragraph below the first paragraph, which remained on the screen. Participants read the scenario at their own pace. With the third keypress, both paragraphs disappeared, and the third screen appeared, presenting a question about a proposed utilitarian action. For example, “Do you obtain the organs cutting the carotid artery of the accident victim, so you can undertake the transplantations for the other five patients?” in the case of doctor and organ transplant scenario (ID 26). Participants judged the moral acceptability of the proposed utilitarian action in each dilemma using a 7-point scale (1 = Completely unacceptable; 7 = Completely acceptable), and their response times were measured, starting from the first screen. The ratings were made by means of keypresses on the number keys. There was no time limit. In the inter-trial interval, a waiting screen was displayed until the participants pressed the spacebar.

The skin temperature of the participants was measured again after the moral dilemma task as the final state (Time 3), at which time the experiment was completed. The participants took about 80min to complete the whole set of experimental procedures. The study was approved by the Ethics Committee of the universities where the data collection was conducted: Senshu University (issue number 16-S001-2) and Hiroshima Shudo University (issue number 2017–10).

### Results

#### Manipulation Assessment

A two-way ANOVA was conducted on the subjective room warmth, comfort, and arousal rating after the habituation phase for each temperature condition (Cool vs. Control vs. Hot) and season (summer vs. winter) as between-subject factors. Bonferroni’s multiple comparison was used for *post hoc* analysis to the entire data set in all statistical analyses except when the requirement of equal error variances was not met. Table 1 shows the means and 95% confidence intervals of room warmth, comfort, and arousal rating and skin temperature at each time of manipulation check in the Cool, Control, and Hot room temperature conditions and the summer and winter seasons. A summary of the results of the main effects of experimental temperature condition in ANOVA of each manipulation check index is shown in Supplementary Table S1. The detailed descriptions of the statistical analyses with respect to the manipulation assessment of subjective indices are presented in the Supplementary Material. In brief, there were significant main effects of temperature condition in room warmth and comfort rating.

**Table 1:** Means and 95% confidence intervals of room warmth rating, comfort rating, arousal rating, skin temperature in the Cool, Control, and Hot temperature conditions, and summer and winter seasons in Experiment 1.

Measures			Cool			Control			Hot		
			Mean	95% CI		Mean	95% CI		Mean	95% CI	
Room warmth	Summer		2.000	1.002	2.998	2.786	2.385	3.187	3.750	3.143	4.357
	Winter		2.813	2.164	3.461	3.923	2.993	4.853	5.200	4.504	5.896
Comfort	Summer		2.333	1.267	3.400	4.857	3.907	5.807	5.500	4.673	6.327
	Winter		3.875	3.057	4.693	4.538	3.603	5.474	5.133	4.417	5.850
Arousal	Summer		3.000	2.002	3.998	3.643	3.023	4.263	2.917	1.862	3.971
	Winter		3.938	3.176	4.699	4.077	3.249	4.905	3.467	2.429	4.504
Skin temp (Celsius)	Summer	Time 1	36.817	36.558	37.075	36.543	36.485	36.601	36.592	36.476	36.707
		Time 2	36.575	36.425	36.725	36.514	36.412	36.617	36.700	36.530	36.870
		Time 3	36.525	36.343	36.707	36.507	36.405	36.609	36.792	36.683	36.900
	Winter	Time 1	37.050	36.841	37.259	36.969	36.632	37.307	36.927	36.605	37.248
		Time 2	36.913	36.684	37.141	36.992	36.718	37.266	37.100	36.807	37.393
		Time 3	36.875	36.686	37.064	36.915	36.675	37.156	36.987	36.796	37.177

**Figure 1:**
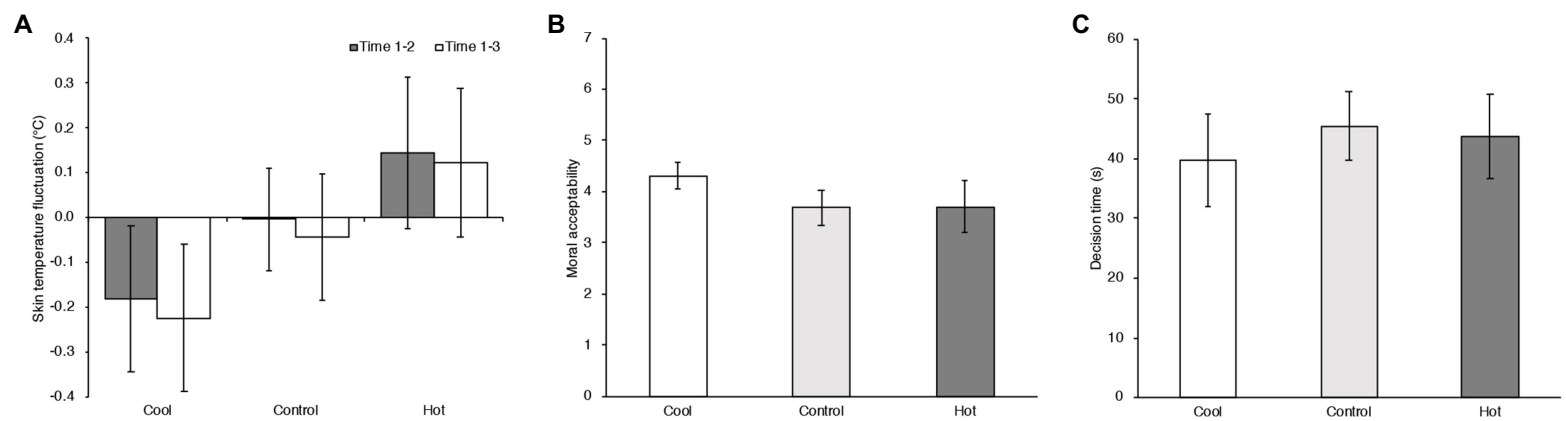
**(A)** Averaged differences in skin temperature between baseline and before the moral dilemma task (Time 1–2; gray bars) and between baseline and after the moral task (Time 1–3; white bars) in Cool, Control, and Hot temperature conditions in Experiment 1. Error bars indicate the 95% confidence intervals of the mean in each condition. **(B)** Mean moral acceptability rating in Cool, Control, and Hot temperature conditions in Experiment 1. Error bars indicate the 95% confidence intervals of the mean in each condition. **(C)** Mean decision times in the experimental conditions in Experiment 1. Error bars indicate the 95% confidence intervals of the mean in each condition.

To assess the effect on physiological indices by temperature manipulation, the amounts of change in skin temperature were calculated by subtracting each value after the experimental task phase (Time 3) and before experimental task phase (Time 2) from that obtained in the baseline phase (Time 1). The indices of skin temperature fluctuation for each temperature condition are shown in Figure 1A. Factorial repeated measure (RM) ANOVA was computed on the skin temperature fluctuation with the within-subject factors measurement time [Before experimental task (Time 1–2) vs. After experimental task (Time 1–3)]. Experimental condition and season were the between-subject factors. A significant main effect of temperature condition was found, *F* (2, 76)=5.825, *MSE*=0.280, *p*=0.004, $\eta_{p}^{2}$=0.133. There was no significant main effect of season, *F* (1, 76)=0.129, *MSE*=0.280, *p*=0.720,$\eta_{p}^{2}$=0.002, nor of the measurement time, *F* (1, 76)=1.124, *MSE*=0.037, *p*=0.293,$\eta_{p}^{2}$=0.015. There was also no significant interaction between temperature condition and season and measurement time, *F* (2, 76)=1.087, *MSE*=0.037, *p*=0.342, $\eta_{p}^{2}$=0.028, nor temperature condition and season, *F* (2, 76)=0.271, *MSE*=0.280, *p*=0.763, $\eta_{p}^{2}$=0.007), nor temperature condition and measurement time, *F* (2, 76)=0.123, *MSE*=0.037, *p*=0.884, $\eta_{p}^{2}$=0.003. *Post hoc* analysis revealed that Hot temperature participants had an increased skin temperature (*M_HOT_*=0.133, 95% CI=[−0.024, 0.291]) as compared to Cool temperature participants (*M_COOL_*=−0.204, 95% CI=[−0.357, −0.050], *t* (79)=3.387, *adj. p*=0.003, Cohen’s *d*=0.901). There was no significant difference between Hot temperature participants and Control temperature participants [*M_CONTROL_*=−0.024, 95% CI=[−0.143, 0.095], *t* (79)=1.568, *adj. p*=0.362, Cohen’s *d*=0.421] nor between Cool temperature participants and Control temperature participants [*t* (79)=1.805, *adj. p*=0.225, Cohen’s *d*=0.480].

Although there was no significant difference between Control and Cool conditions, the subjective warmth perception was manipulated according to the experimental conditions. There were also no significant differences between the Control condition and the two experimental conditions with respect to skin temperature fluctuation; however, the trends of variation matched the experimental settings. These results indicated that the experimental room temperature manipulation was performed effectively from both cognitive and physiological perspectives.

#### Moral Judgment

Figures 1B,C shows the means and 95% confidence intervals of the moral judgment ratings and decision times in the Cool, Control, and Hot room temperature conditions. Variation from differences of stimuli in the experiment may be considered as a random effect for analysis (Judd et al., 2012). Therefore, a generalized linear mixed effect model with Poisson distribution was computed to treat the 16 scenarios of the moral dilemma task as a random effect, using the lme4 package (Bates et al., 2015) in the R environment (Ver. 3.6.0; R Core Team, 2019). The moral judgment rating was a discrete variable, with integers from 1 to 7. Since this dependent variable reflects the frequency of events, we applied the Poisson distribution rather than the Gaussian distribution for the analysis. For other dependent variables (not based on integers or including negative values), we applied the Gaussian distribution. Temperature condition, each scenario factor in the dilemma task (Personal Force, Benefit Recipient, Evitability), and the interaction between the temperature condition and scenario factors up to three-way (e.g., temperature condition × Personal Force × Evitability) were modeled as fixed effects. Because there was a significant interaction between experimental condition and season on the indices of the manipulation check, season and interaction of season and experimental condition were modeled as fixed effects to rule out effects of such an interaction. Moreover, comfort ratings were different as a function of the temperature condition. There is a possibility that the temperature effect can be accounted for by comfort feeling rather than temperature setting. To examine this point, the comfort rating was also modeled as a fixed effect. Finally, the participants and stimuli (dilemma scenarios) were modeled as random effects.

Categorical variables, Season (Summer/Winter), Personal Force (Personal harm/Impersonal harm), Benefit recipient (Other-Beneficial/Self-Beneficial), and Evitability (Avoidable harm/Inevitable harm) were coded as −0.5/+0.5 contrasts. Temperature condition was coded as a combination of two categorical variables C1 and C2 with −0.5/+0.5 contrasts. The Control condition was coded with C1: −0.5 and C2: −0.5; the Cool condition with C1: +0.5 and C2: −0.5; and the Hot condition with C1: −0.5 and C2: +0.5.

Variance of the random effects, regression coefficients (*b*) and 95% confidence intervals of the fixed effects, *z*-values and *p*-values are shown in Table 2. The main effect of Cool temperature condition (C1) was significant (*b*=0.193, 95% CI=[0.026, 0.360]). Cool temperature increased utilitarian judgment as compared to the other two conditions. Conversely, there was no significant effect of the Hot temperature condition (C2: *b*=−0.008, 95% CI=[−0.160, 0.142]). There was no significant interaction between temperature condition and season (C1: *b*=−0.087, 95% CI=[−0.400, 0.227]; C2: *b*=−0.154, 95% CI=[−0.449, 0.141]) nor between temperature condition and the three scenario factors: Personal Force and temperature condition (C1: *b*=0.022, 95% CI=[−0.116, 0.159]; C2: *b*=0.061, 95% CI=[−0.080, 0.201]); Benefit Recipient and temperature condition (C1: *b*=0.114, 95% CI=[−0.025, 0.254]; C2: *b*=0.035, 95% CI=[−0.107, 0.177]); and Evitability and temperature condition (C1: *b*=−0.085, 95% CI=[−0.225, 0.054]; C2: *b*=−0.020, 95% CI=[−0.162, 0.122]). Comfort rating also had no effect (*b*=−0.002, 95% CI=[−0.044, 0.041]).

**Table 2:** Summary of the results from the generalized linear mixed effect model in Experiment 1.

Effect	Variance	*b*	95% CI		*z*	Value of *p*
**Fixed effects**						
Intercept		1.341^***^	1.126	1.553	12.451	< 0.001
Cool temperature (C1)		0.193^*^	0.026	0.360	2.294	0.022
× Season		−0.087	−0.400	0.227	−0.551	0.581
× Personal force		0.022	−0.116	0.159	0.311	0.756
× Benefit recipient		0.114	−0.025	0.254	1.619	0.105
× Evitability		−0.085	−0.225	0.054	−1.209	0.227
Hot temperature (C2)		−0.008	−0.160	0.142	−0.102	0.918
× Season		−0.154	−0.449	0.141	−1.035	0.301
× Personal force		0.061	−0.080	0.201	0.853	0.394
× Benefit recipient		0.035	−0.107	0.177	0.482	0.630
× Evitability		−0.020	−0.162	0.122	−0.274	0.784
Personal force		0.147	−0.051	0.347	1.539	0.124
Benefit recipient		0.096	−0.102	0.296	1.002	0.316
Evitability		0.242^*^	0.044	0.442	2.528	0.011
Season		−0.123	−0.278	0.031	−1.590	0.112
Comfort		−0.002	−0.044	0.041	−0.071	0.943
**Random effects**						
Participants						
Intercept	0.056					
Stimuli						
Intercept	0.032					

* *=p<0.05*;

**=p<0.01;

*** *=p<0.001*.

*b = regression coefficients (not standardized); the range of the outcome variable was from 1 to 7*.

The results showed a temperature effect on moral judgment. Previous studies reported that decision time was associated with moral judgment tendency in the moral dilemma task such that longer decision correlated with utilitarian judgment (Greene et al.,2001; Suter and Hertwig, 2011). To examine whether temperature affected the decision time here, we computed a generalized linear mixed effect model with Gaussian distribution. The temperature conditions (C1, C2) were modeled as fixed effects. The participants and stimuli were modeled as random effects. The results showed no significant effect, neither for the Cool temperature condition (C1: *b*=−5.810, 95% CI=[−15.129, 3.509], *t* (79)=−1.217, *p*=0.227) nor for the Hot temperature condition (C2: *b*=−1.758, 95% CI=[−11.161, 7.646], *t* (79)=−0.365, *p*=0.716). Thus, the temperature manipulation only affected the moral judgment ratings.

### Discussion

As in the previous study (Nakamura et al., 2014), temperature impacted on the moral dilemma decision-making, with enhanced utilitarian judgment in the Cool condition. Moreover, while the original study found the temperature effect was mediated by the scenario factor, the present study observed a stronger effect that was not limited to the impersonal dilemma situations. Ambient temperature manipulation might be more effective than manipulation through the sense of touch. On the other hand, no contrasting effect was found for Hot temperature compared to Cold.

## Experiment 2

In Experiment 1, we found that environmental temperature affects moral judgment. Our findings raised the possibility that ambient manipulations of temperature have a bigger impact on moral judgment than haptic manipulations. Experiment 2 was carried out to provide empirical evidence on this point.

### Method

#### Participants

The a priori sample size calculation was conducted based on the result of Experiment 1, specifically, based on the main effect of the temperature condition on moral judgment in the ANOVA of Experiment 1 [*F* (2, 76)=3.643, *MSE*=7.650, *p*=0.031,$\eta_{p}^{2}$=0.087], with power of 0.80, alpha of 0.05, and effect size f of 0.309. The calculation indicated a minimum sample size of 20 per group. To compare with Experiment 1, we determined to collect at least 27 participants per group: same as in Experiment 1. Participants were 82 healthy Japanese undergraduate and graduate students (30 males and 52 females, age: *M_age_*=18.56, *SD_age_*=0.80). Participants were randomly assigned to one of three different temperature conditions: Control (11 males, 17 females), 1 part cooled (6 males, 21 females), 3 parts cooled (13 males, 14 females). The number of participants in each condition was unbalanced due to unexpected cancellation. The present experiment was conducted in June, July and October. All participants were naïve to the purpose of the experiment. Each participant received compensation worth 1,000 yen for their participation. Written informed consent was obtained from all participants before the experiment.

#### Apparatus

The apparatus was as in Experiment 1.

### Measures

The measures were almost the same as in Experiment 1 except that new questions were added to the subjective measurements. Besides room warmth, questions on body warmth, forehead warmth, neck warmth and hand warmth were added to check that the experimental manipulation was conducted effectively. These items were measured by 7-point scale (1 = very cool; 7 = very warm).

### Procedure

The flow of the procedure was almost the same as in Experiment 1. The participants were explained the experimental procedure in the experiment room for about 10min at first. The experimental room temperature was kept at 24°C (Control: *M_temperature_*=23.99, *SD_temperature_*=0.23; 1 part: *M_temperature_*=23.99, *SD_temperature_*=0.26; 3 parts: *M_temperature_*=24.12, *SD_temperature_*=0.27); this was the same temperature as in the Control condition in Experiment 1, operated through the room air conditioner with circulator. After the instruction, the skin temperature of the participant’s right hand was measured as the baseline (Time 1) of their physiological level. Then, three thermal pads (Hot and Cool Pad size S, Fujisho Incorporation, Japan) were placed on the forehead, neck, and left hand. In the Control condition, all three pads were not cooled and left at room temperature. In the 1-part condition, only the pad for the neck was cooled and the others were not cooled. In the 3-part condition, all three pads were cooled. Cooled pads were chilled in the freezer for at least 4hours. First, the participants waited for 5min alone in the experiment room while relaxing to habituate to the experimental environment. After the habituation period, they filled out the questionnaire about the subjective warmth and feelings of their comfort and arousal; also, their skin temperature was measured before the moral dilemma task as a manipulation check (Time 2). They were asked to perform the moral dilemma task after the manipulation check. The participants judged the moral acceptability of the proposed utilitarian action in each dilemma using a 7-point scale (1 = Completely unacceptable; 7 = Completely acceptable), and their response times were measured, starting from the first screen. The ratings were made by mouse click. There was no time limit. The skin temperature of the participants was measured again after the moral dilemma task as the final state (Time 3), at which time the experiment was completed. The participants took about 45min to complete the whole set of experimental procedures. The study was approved by Hiroshima Shudo University’s Ethics Committee (issue number 2018–0003).

### Results

#### Manipulation Assessment

A one-way ANOVA was conducted on the subjective room warmth, body warmth, forehead warmth, neck warmth, hand warmth, comfort, and arousal rating after the habituation phase for each manipulation condition (Control vs. 1 part vs. 3 parts) as a between-subject factor. Bonferroni’s multiple comparison was used for *post hoc* analysis to the entire data set in all statistical analyses except when the assumption of equality of error variances was violated. Table 3 shows the means and 95% confidence intervals of room warmth, body warmth, forehead warmth, neck warmth, hand warmth, comfort, and arousal rating and skin temperature at each time of manipulation check among the Control, 1-part, and 3-part manipulation conditions. A summary of the results of the main effects of the experimental temperature condition in the ANOVA of each manipulation check index is shown in Supplementary Table S2. The detailed descriptions of the statistical analyses with respect to the manipulation assessment of subjective indices are presented in the Supplementary Material. In brief, there were significant main effects of temperature condition in all indices except for room warmth rating.

**Table 3:** Means and 95% confidence intervals of room warmth rating, body warmth rating, forehead warmth rating, neck warmth rating, hand warmth rating, comfort rating, arousal rating, and skin temperature in the Control, 1 part, and 3 parts conditions in Experiment 2.

Measures		Control			1 part			3 parts		
		Mean	95% CI		Mean	95% CI		Mean	95% CI	
Warmth	Room	3.321	2.956	3.687	3.259	2.920	3.598	3.037	2.666	3.408
	Body	3.786	3.360	4.212	3.185	2.821	3.549	2.407	2.093	2.722
	Forehead	3.750	3.361	4.139	4.111	3.777	4.446	1.963	1.707	2.219
	Neck	4.000	3.635	4.365	2.333	1.909	2.757	2.370	2.021	2.719
	Hand	3.821	3.456	4.187	4.222	3.926	4.519	1.889	1.573	2.205
Comfort		4.857	4.366	5.348	4.519	4.037	5.001	3.963	3.505	4.421
Arousal		4.143	3.663	4.622	3.407	3.009	3.806	4.259	3.827	4.692
Skin temp (Celsius)	Time 1	36.275	36.001	36.549	36.363	36.122	36.603	36.381	36.158	36.605
	Time 2	36.468	36.303	36.633	36.404	36.189	36.619	36.430	36.255	36.604
	Time 3	36.329	36.077	36.580	36.248	35.934	36.562	35.870	35.511	36.229

**Figure 2:**
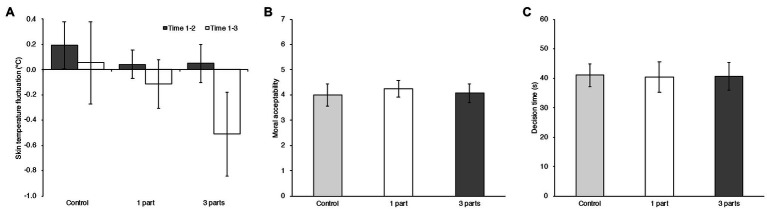
**(A)** Averaged differences in skin temperature between baseline and before the moral dilemma task (Time 1–2; gray bars) and between baseline and after the moral task (Time 1–3; white bars) in the Control, 1-part, and 3-part conditions in Experiment 2. Error bars indicate the 95% confidence intervals of the mean in each condition. **(B)** Mean moral acceptability rating in Control, 1-part, and 3-part conditions in Experiment 2. Error bars indicate the 95% confidence intervals of the mean in each condition. **(C)** Mean decision times in the three conditions in Experiment 2. Error bars indicate the 95% confidence intervals of the mean in each condition.

To assess the effect on physiological indices by temperature manipulation, the amounts of change in skin temperature were calculated by subtracting each value after the experimental task phase (Time 3) and before experimental task phase (Time 2) from that obtained in the baseline phase (Time 1). The indices of skin temperature fluctuation for each manipulation area are shown in Figure 2A. Factorial RM ANOVA was computed on the skin temperature fluctuation with the within-subject factor measurement time [Before experimental task (Time 1–2) vs. After experimental task (Time 1–3)]. Temperature condition was the between-subject factor. A significant main effect of temperature condition was found, *F* (2, 79)=3.279, *MSE*=0.529, *p*=0.043, $\eta_{p}^{2}$=0.077, and a significant main effect of Measurement time, *F* (1, 79)=18.549, *MSE*=0.179, *p*<0.001,$\eta_{p}^{2}$=0.190. There was a significant interaction effect between temperature condition and measurement time, *F* (2, 79)=4.296, *MSE*=0.179, *p*=0.017, $\eta_{p}^{2}$=0.098. In a simple main effect analysis, the effect of temperature condition proved to be significant in Time 1–3, *F* (2, 79)=4.138, *MSE*=0.556, *p*=0.020,$\eta_{p}^{2}$=0.095. Post-hoc analysis revealed that the skin temperature decreased more strongly in participants in the 3 parts condition (*M_3 PARTS_*=−0.511, 95% CI=[−0.843, −0.179]) than those in the Control condition (*M_CONTROL_*=0.054, 95% CI=[−0.274, 0.381], *t* (79)=2.809, *adj. p*=0.019, Cohen’s *d*=1.066). There was no significant difference between participants in the 3-part condition and those in the 1 part condition (*M_1 PART_*=−0.115, 95% CI=[−0.308, 0.078], *t* (79)=1.954, *adj. p*=0.163, Cohen’s *d*=0.748), nor between participants in the 1 part condition and those in the Control condition [*t* (79)=0.838, *adj. p*=1.000, Cohen’s *d*=0.318]. The effect of measurement time was also significant in the 3 parts condition, *F* (1, 79)=23.575, *MSE*=0.179, *p*<0.001,$\eta_{p}^{2}$=0.230. Compared with the mean skin temperature fluctuation for Time 1–2 (*M_TIME1-2_*=0.048, 95% CI=[−0.102, 0.198]), the skin temperature for Time 1–3 decreased more strongly (*M_TIME1-3_*=−0.511, 95% CI=[−0.843, −0.179]).

Overall, the manipulation checks showed significant subjective perceptions of cold only in the terms of body or body parts, not in terms of room warmth, confirming that the haptic manipulation of temperature was successful and distinct from ambient temperature. Skin temperature decreased significantly in participants in the 3-part condition as compared to those in the Control condition. Thus, the haptic manipulation was also successful from a physiological perspective, specifically in the 3 parts condition.

#### Moral Judgment

The means and 95% confidence intervals of the moral judgment ratings and decision times in the Control, 1-part, and 3-part manipulation conditions are shown in Figures 2B,C. A generalized linear mixed effect model with Poisson distribution was computed. Experimental condition, each scenario factor in the dilemma task (Personal Force, Benefit Recipient, Evitability), comfort rating and interaction between temperature condition and scenario factors up to three-way were modeled as fixed effects. The participants and stimuli (dilemma scenarios) were modeled as random effects. Categorical variables, Personal Force (Personal harm/Impersonal harm), Benefit recipient (Other-Beneficial/Self-Beneficial), and Evitability (Avoidable harm/Inevitable harm) were coded as −0.5/+0.5 contrasts, same as Experiment 1. Experimental condition was coded as a combination of two categorical variables C1 and C2 with −0.5/+0.5 contrasts. The Control condition coded with C1: −0.5 and C2: −0.5; the 1-part manipulation condition with C1: +0.5 and C2: −0.5; and the 3 parts manipulation condition with C1: −0.5 and C2: +0.5.

Variance of the random effects, regression coefficients (*b)* and 95% confidence intervals of the fixed effects, *z*-values, and *p*-values are shown in Table 4. There was no significant effect of the temperature condition, neither for the 1 part manipulation condition (C1: *b*=0.067, 95% CI=[−0.064, 0.198]) nor for the 3 parts manipulation condition (C2: *b*=0.021, 95% CI=[−0.116, 0.157]). There was also no significant interaction between Experimental condition and any of the three scenario factors: Personal Force and temperature condition (C1: *b*=−0.030, 95% CI=[−0.161, 0.101]; C2: *b*=−0.009, 95% CI=[−0.141, 0.124]); Benefit Recipient and temperature condition (C1: *b*=−0.045, 95% CI=[−0.177, 0.087]; C2: *b*=−0.042, 95% CI=[−0.176, 0.091]); and Evitability and temperature condition (C1: *b*=−0.066, 95% CI=[−0.198, 0.067]; C2: *b*=−0.036, 95% CI=[−0.169, 0.098]). Comfort rating also had no effect (*b*=0.000, 95% CI=[−0.044, 0.045]).

**Table 4:** Summary of the results from the generalized linear mixed effect model in Experiment 2.

Effect	Variance	*b*	95% CI		*z*	Value of *p*
**Fixed effects**						
Intercept		1.375^***^	1.160	1.589	12.688	< 0.001
1 part manipulation (C1)		0.067	−0.064	0.198	1.014	0.310
× Personal force		−0.030	−0.161	0.101	−0.449	0.654
× Benefit recipient		−0.045	−0.177	0.087	−0.672	0.502
× Evitability		−0.066	−0.198	0.067	−0.976	0.329
3 parts manipulation (C2)		0.021	−0.116	0.157	0.298	0.766
× Personal force		−0.009	−0.141	0.124	−0.130	0.896
× Benefit recipient		−0.042	−0.176	0.091	−0.623	0.534
× Evitability		−0.036	−0.169	0.098	−0.524	0.600
Personal force		0.121	−0.064	0.308	1.356	0.175
Benefit recipient		0.045	−0.140	0.232	0.503	0.615
Evitability		0.171	−0.014	0.358	1.914	0.056
Comfort		0.000	−0.044	0.045	0.021	0.983
**Random effects**						
Participants						
Intercept	0.043					
Stimuli						
Intercept	0.027					

*=p<0.05;

**=p<0.01;

*** *=p<0.001*.

*b = regression coefficients (not standardized); the range of the outcome variable was from 1 to 7*.

To examine the temperature effect on the decision-making process, a generalized linear mixed effect model with Gaussian distribution was computed using the decision times as dependent variable. The experimental conditions (C1, C2) were modeled as fixed effects. The participants and stimuli were modeled as random effects. The results showed no significant effects, neither for the 1-part manipulation condition (C1: *b*=−0.437, 95% CI=[−6.407, 5.533], *t* (78)=−0.143, *p*=0.887), nor for the 3-part manipulation condition (C2: *b*=−0.419, 95% CI=[−6.448, 5.609], *t* (78)=−0.136, *p*=0.892).

### Discussion

Experiment 2 examined the effect of haptic cold temperature on moral judgment. However, there was no cool temperature effect in any aspect of moral judgment. This amounts to a failure of replication of the haptic temperature effect observed by Nakamura et al. (2014), who asked participants to wear a scarf with frozen internal water packs. The absence of a haptic cool temperature effect on moral judgment in our data occurred despite the fact that our manipulations proved successful in eliciting both a subjective sense of cold and a physiological measure of temperature reduction. Our findings in Experiment 2 indicate that the haptic temperature manipulation could not reproduce the cool ambient temperature effect we observed in Experiment 1.

There is no obvious reason why there should be a privileged pathway from ambient temperature toward an influence on moral judgment. None of the psychological or physiological accounts of temperature effects on higher-order social cognition offer any insights in this regard. A more critical reading of our data from Experiments 1 and 2 might simply argue that we have conflicting evidence, suggesting that the effects of temperature on moral judgment are weak or have limited reproducibility. To gain further insights into the phenomenon, we opted to examine its generalizability (or lack thereof).

## Experiment 3

Ambient cold temperature had an effect on moral judgment in Experiment 1, but haptic temperature did not in Experiment 2. We endeavored to examine the generalizability of the ambient cool temperature effect found in Experiment 1 by comparing it with another moral judgment paradigm. In Experiment 3, we asked participants to perform the moral dilemma task used in Experiments 1 and 2, but we also conducted a moral acceptability judgment task (“the moral image task”) with visual images of real-world scenes. The moral image task included situations depicting matters of life or death as well as less dramatic images; this was in contrast with the situation of the moral dilemma task, which always presented matters of life or death. There were no dilemmas or choices about life or death presented in the moral image task. Moreover, the moral image task only required subjects to judge the moral acceptability of the situation, whereas the judgments in the moral dilemma task required the subjects to specify their own action to resolve the situation.

If the ambient cool temperature properly affects moral decision-making, inducing participants to be more “cold-hearted” or “cool-headed,” significant effects from temperature should be obtained in both moral judgment tasks. If ambient cool temperature affects only the thinking in terms of sacrificial behavior toward the greater good, then significant effects from temperature should be obtained only in the moral dilemma task.

### Method

#### Participants

Sample size was determined by conducting an a-priori sample size calculation, with the same parameters as in Experiment 2. Since the effect size on the moral image task was unknown, we calculated the sample size based on the moral dilemma task, with effect size f of 0.309 which was from the result of Experiment 1. The calculation indicated a minimum sample size of 25 participants per temperature condition. In order to control the sample size with a view to counterbalancing the order of tasks, the sample size in each group should be an even number. Therefore, we initially aimed to work with four groups of 14 participants, for a total sample size of 56.

Due to recruitment constraints, we were able to collect data from 48 healthy Japanese undergraduate and graduate students (24 males and 24 females, age: *M_age_*=21.29, *SD_age_*=2.53). Participants were randomly assigned to one of the two different room temperature conditions: 26°C as Control (12 males, 12 females), and 21°C as Cool (12 males, 12 females). It should be mentioned that the actual number of participants did not meet our criteria of sample size. However, with 24 instead of 25 participants per condition (as indicated by a-priori sample size calculation), we propose that the current sample size is still large enough to warrant careful consideration. The present study was conducted in July and August. All subjects were naïve to the purpose of the experiment. Each subject received compensation worth 1,000 yen for their participation. Written informed consent was obtained from all participants before the experiment.

#### Apparatus

A desktop computer with Psychopy software (version 1.90.3) with PyTribe library controlled all events and data collection. All visual stimuli were presented on a 23.8-inch monitor, with a display resolution of 1920×1080 pixels. To minimize the head movement by participants and to control the distance to the screen, a chin-rest with a forehead-support was used. The monitor screen was set approximately 62cm from the chin-rest. The evaluation responses in the tasks were recorded using a joystick (Model no. 963290–0403, Logitech, Switzerland).

### Measures

The measures of the indices for the subjective and physiological manipulation checks were the same as in Experiment 1, except that the question of body warmth was added to the subjective measurements.

#### Moral Dilemma Task

The materials and procedures were the same as for the moral dilemma task in Experiment 1, except that the rating scale was changed from 7-point scale to a continuous rating scale from −10 to 10 (−10 = Completely unacceptable; 10 = Completely acceptable), in line with previous value-based decision-making paradigms performed in our laboratory (Ounjai et al., 2018; Wolf et al., 2018).

#### Moral Image Task

Sixty visual stimuli were selected from the Socio-Moral Image Database (SMID) developed by Crone et al. (2018). This database provided the largest standardized moral stimulus set, covering a wide range of morally positive, negative and neutral content. The database offered the norming values of all stimuli from arousal, authority, fairness, harm, ingroup, moral, purity, and valence perspective by 5-point scale. Two sets of images were selected from the database for inclusion in the present study using the genetic algorithm stimuli sampling recommended Crone et al. (controlling for parameters other than the moral valence). One set of 30 images (designated “moral images”) was composed of images with an SMID valence rating of higher than 3.5. The second set of 30 images (designated “immoral images”) consisted of images with an SMID valence rating lower than 2.5. Participants were asked to judge the moral acceptability of these images on a continuous rating scale from −10 to 10 (−10 = Completely unacceptable; 10 = Completely acceptable). The images were presented in random order.

### Procedure

Participants were tested individually. At the beginning of the experiment, they were given an explanation about the experimental procedure in the experiment room for about 10min. The room temperature was controlled based on each experimental condition, Cool (*M_temperature_*=21.18, *SD_temperature_*=0.25) vs. Control (*M_temperature_*=26.19, *SD_temperature_*=0.13), operated through the room air conditioner with circulator. Informed consent was obtained from each participant during this period.

After the instruction, the skin temperature of the participant’s forehead was measured as the baseline (Time 1) of their physiological level. First, the participants waited for 10min in the experiment room while relaxing to habituate to the experimental environment. Practice trials for the joystick rating response were conducted during this time. After the habituation period, the participants filled out the questionnaire about the subjective warmth and feelings of their comfort and arousal; also, their skin temperature was measured before the first moral judgment task as a manipulation check (Time 2). The participants were asked to perform two kinds of moral judgment tasks after the manipulation check. The task order for each participant was counterbalanced.

In the moral image task, the first screen presented a fixation cross at the center for 1s. The participants were asked to gaze at the fixation cross until a stimulus appeared. After the fixation, the second screen presented a stimulus with a height of 400 pixels. The participants viewed the stimulus at their own pace. The second screen disappeared upon pressing the spacebar and was replaced by the third screen, presenting the question: “How morally acceptable is this picture?.” The participants judged the moral acceptability of each stimulus on a continuous rating scale from −10 to 10 by moving the joystick to a position on the scale and pulling the trigger. Response times were measured. There was no time limit. In the inter-trial interval, a waiting screen was displayed until the participants pressed the spacebar.

The flow of the moral dilemma task was similar to that in the moral image task. First, the participants were asked to gaze at a fixation cross. After the fixation, the next two screens described the dilemma scenario. The screen that contained the dilemma scenario disappeared upon pressing the spacebar, and the fourth screen appeared and presented a question about a proposed utilitarian action. The participants judged the moral acceptability of the proposed utilitarian action for each dilemma using the same rating scale as in the image task, and the response times were measured.

The skin temperature of the participants was measured again after the two moral judgment tasks as the final state (Time 3), at which time the experiment was completed. The participants took about 60min to complete the whole set of experimental procedures. The study was conducted in accordance with the ethical principles of Kyushu University and approved by the Human Ethics Committee of the Faculty of Arts and Science (issue number 201902).

### Results

#### Manipulation Assessment

Welch’s independent *t*-test was computed on the subjective room warmth, body warmth, comfort, and arousal rating after the habituation phase for each temperature condition (Control vs. Cool) as the between-subject factor. Table 5 shows the means and 95% confidence intervals of room warmth, body warmth, comfort, and arousal rating, and skin temperature at each time of manipulation check in the Control and Cool temperature conditions. A summary of the results of the main effects of experimental temperature condition in t-tests of each manipulation check index is shown in Supplementary Table S3. The detailed descriptions of the statistical analyses with respect to the manipulation assessment of subjective indices are presented in the Supplementary Material. In brief, there was a significant main effect of temperature condition in room warmth, body warmth, and comfort rating.

**Table 5:** Means and 95% confidence intervals of room warmth rating, body warmth rating, comfort rating, arousal rating, and skin temperature in the Control and Cool temperature conditions in Experiment 3.

Measures		Control			Cool		
		Mean	95% CI		Mean	95% CI	
Warmth	Room	3.958	3.695	4.221	2.958	2.642	3.275
	Body	4.375	4.028	4.722	3.333	2.968	3.699
Comfort		5.333	4.874	5.793	4.333	3.680	4.987
Arousal		4.042	3.603	4.481	4.375	3.782	4.968
Skin temp (Celsius)	Time 1	36.567	36.480	36.653	36.700	36.601	36.799
	Time 2	36.638	36.559	36.716	36.704	36.605	36.803
	Time 3	36.654	36.571	36.737	36.729	36.654	36.804

**Figure 3:**
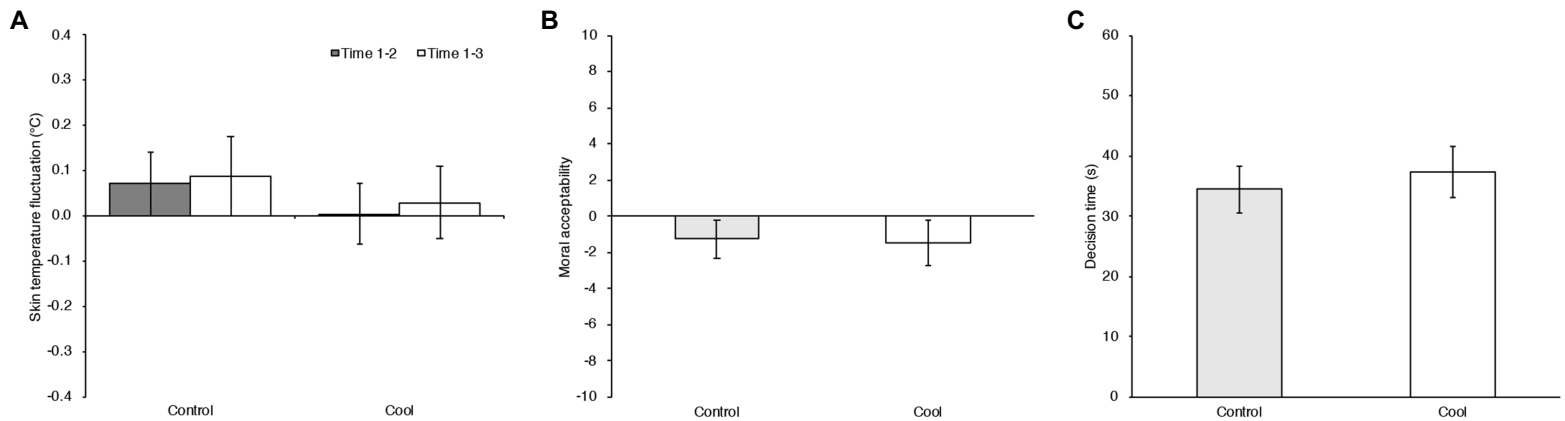
**(A)** Averaged differences in skin temperature between baseline and before the moral judgment tasks (Time 1–2; gray bars) and between baseline and after the moral judgment tasks (Time 1–3; white bars) in the Control and Cool temperature conditions in Experiment 3. Error bars indicate the 95% confidence intervals of the mean in each condition **(B)** Mean moral acceptability rating of moral dilemma task in Control and Cool temperature conditions in Experiment 3. Error bars indicate the 95% confidence intervals of the mean in each condition **(C)** Mean decision times of the moral dilemma task in each condition in Experiment 3. Error bars indicate the 95% confidence intervals of the mean in each condition.

To assess the effect on physiological indices by temperature manipulation, the amounts of change in skin temperature were calculated by subtracting each value after the experimental task phase (Time 3) and before the experimental task phase (Time 2) from that obtained in the baseline phase (Time 1). The indices of skin temperature fluctuation for each temperature condition are shown in Figure 3A. Factorial RM ANOVA was computed on the skin temperature fluctuation with the within-subject factor measurement time (Before experimental task [Time 1–2) vs. After experimental task (Time 1–3)]. The Temperature condition was the between-subject factor. There was no significant main effect of temperature condition, *F* (1, 46)=1.770, *MSE*=0.053, *p*=0.190, $\eta_{p}^{2}$=0.037, nor of measurement time, *F* (1, 46)=0.800, *MSE*=0.013, *p*=0.376,$\eta_{p}^{2}$=0.017. There was also no significant effect interaction between temperature condition and measurement time, *F* (1, 46)=0.032, *MSE*=0.013, *p*=0.859, $\eta_{p}^{2}$=0.001.

These results indicated that the subjective state was manipulated effectively as in Experiment 1. However, the experimental manipulation did not affect the physiological measures.

#### Moral Judgment in the Dilemma Task

The means and 95% confidence intervals of the moral judgment ratings and decision times in the Control and Cool room temperature conditions are shown in Figures 3B,C. A generalized linear mixed effect model with Gaussian distribution was computed. Temperature condition, each scenario factor in dilemma task (Personal Force, Benefit Recipient, Evitability), and the interaction between temperature condition and scenario factors up to three-way (e.g., Temperature condition × Personal Force × Evitability) were modeled as fixed effects. There is a possibility that the two moral judgment tasks affect each other. The duration of exposure to temperature can also be factor. Therefore, Task order and the interaction of Task order and Temperature condition were modeled as fixed effects. Comfort rating was also modeled as a fixed effect to control for any mediation on the temperature manipulation. The participants and stimuli (dilemma scenarios) were modeled as random effects. Categorical variables, Temperature condition (Control/Cool), Task order (Dilemma task first/Dilemma task second), Personal Force (Personal harm/Impersonal harm), Benefit recipient (Other-Beneficial/Self-Beneficial), and Evitability (Avoidable harm/Inevitable harm) were coded as −0.5/+ 0.5 contrasts.

Variance of the random effects, regression coefficients (*b*) and 95% confidence intervals of the fixed effects, *t*-values and *p*-values are shown in Table 6. There was no significant effect of Cool temperature condition (*b*=−0.760, 95% CI=[−2.372, 0.851]). However, a significant effect of the interaction between Temperature condition and Task order was found (*b*=3.421, 95% CI=[0.304, 6.537]). Simple slope analyses to follow up on this interaction did not produce any significant effects. There was no significant interaction between Temperature condition and any of the three scenario factors: Personal Force and temperature condition (*b*=0.229, 95% CI=[−1.040, 1.498]); Benefit Recipient and temperature condition (*b*=0.303, 95% CI=[−0.966, 1.572]); and Evitability and temperature condition (*b*=−0.210, 95% CI=[−1.479, 1.060]). Comfort rating also had no effect (*b*=−0.524, 95% CI=[−1.143, 0.096]).

**Table 6:** Summary of the results from the generalized linear mixed effect model of the moral dilemma task in Experiment 3.

Effect	Variance	*b*	95% CI		*t*	*df*	Value of *p*
**Fixed effects**							
Intercept		1.160	−2.014	4.333	0.680	49.369	0.499
Cool temperature		−0.760	−2.372	0.851	−0.898	43.000	0.083
× Order		3.421^*^	0.304	6.537	2.089	43.000	0.043
× Personal force		0.229	−1.040	1.498	0.352	699.000	0.725
× Benefit recipient		0.303	−0.966	1.572	0.467	699.000	0.641
× Evitability		−0.210	−1.479	1.060	−0.323	699.000	0.747
Personal force		1.543	−0.196	3.281	1.405	9.000	0.194
Benefit recipient		−0.153	−1.891	1.586	−0.139	9.000	0.892
Evitability		3.577^*^	1.838	5.315	3.257	9.000	0.010
Order		0.656	−0.903	2.214	0.801	43.000	0.428
Comfort		−0.524	−1.143	0.096	−1.609	43.000	0.115
**Random effects**							
Participants							
Intercept	6.065						
Stimuli							
Intercept	4.402						

* *=p<0.05*;

**=p<0.01;

***=p<0.001.

*b = regression coefficients (not standardized); the range of the outcome variable was from −10 to 10*.

To examine the temperature effect on the decision-making process, a generalized linear mixed effect model with Gaussian distribution was computed using the decision times. Temperature condition and Task order and interaction between Temperature condition and Task order were modeled as fixed effects. The participants and stimuli were modeled as random effects. The results showed no significant effect, neither from temperature condition (*b*=2.875, 95% CI=[−2.373, 8.123], *t* (44)=1.056, *p*=0.297) nor from task order (*b*=−5.297, 95% CI=[−10.545, −0.049], *t* (44)=−1.946, *p*=0.058). There was no significant interaction between Temperature condition and Task order (*b*=0.628, 95% CI=[−9.868, 11.124], *t* (44)=0.115, *p*=0.909).

#### Moral Judgment in the Image Task

**Figure 4:**
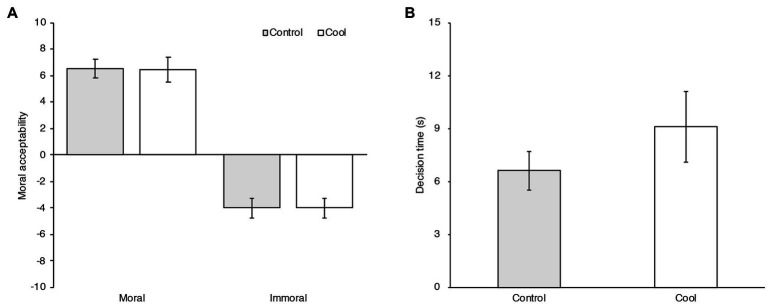
**(A)** Mean moral acceptability ratings in the moral image task in Control and Cool temperature conditions in Experiment 3. Error bars indicate the 95% confidence intervals of the mean in each condition. **(B)** Mean decision times in the moral image task in each condition in Experiment 3. Error bars indicate the 95% confidence intervals of the mean in each condition.

The means and 95% confidence intervals of the moral judgment ratings and decision times in the Control and Cool room temperature conditions are shown in Figures 4A,B. A generalized linear mixed effect model with Gaussian distribution was computed. Temperature condition, Stimuli type in the image task, and interaction between Temperature condition and Stimuli type were modeled as fixed effects. Task order and comfort rating, and the interaction of Task order and Temperature condition were modeled as in the dilemma task. The participants and stimuli (moral images) were modeled as random effects. Categorical variables, Temperature condition, Task order, and Stimuli type (Immoral/Moral) were coded as −0.5/+ 0.5 contrasts.

Variance of the random effects, regression coefficients (*b*), and 95% confidence intervals of the fixed effects, *t*-values and *p*-values are shown in Table 7. There was no significant effect of Cool temperature condition (*b*=−0.037, 95% CI=[−0.915, 0.841]). There was no significant interaction between Temperature condition and Task order (*b*=−0.527, 95% CI=[−2.225, 1.171]) nor between Temperature condition and Stimuli type (*b*=−0.056, 95% CI=[−0.575, 0.463]). Comfort rating also had no effect (*b*=−0.008, 95% CI=[−0.346, 0.329]).

**Table 7:** Summary of the results from the generalized linear mixed effect model of the moral image task in Experiment 3.

Effect	Variance	*b*	95% CI		*t*	*df*	Value of *p*
**Fixed effects**							
Intercept		1.270	−0.468	3.008	1.391	48.993	0.171
Cool temperature		−0.037	−0.915	0.841	−0.080	43.001	0.937
× Order		−0.527	−2.225	1.171	−0.591	43.001	0.558
× Stimuli type		−0.056	−0.575	0.463	−0.212	2772.000	0.832
Stimuli type		10.469^***^	9.517	11.420	21.610	58.000	< 0.001
Order		−0.646	−1.495	0.203	−1.448	43.001	0.155
Comfort		−0.008	−0.346	0.329	−0.047	43.001	0.962
**Random effects**							
Participants							
Intercept	1.963						
Stimuli							
Intercept	3.257						

*=p<0.05;

**=p<0.01;

*** *=p<0.001*.

*b = regression coefficients (not standardized); the range of the outcome variable was from −10 to 10*.

To examine the temperature effect on the decision-making process, a generalized linear mixed effect model with Gaussian distribution was computed using the decision times. Temperature condition and Task order, and interaction between Temperature condition and Task order were modeled as fixed effects. The participants and stimuli were modeled as random effects. The results showed a significant effect of Temperature condition (*b*=2.472, 95% CI=[0.381, 4.564], *t* (44)=2.267, *p*=0.028). Cool temperature condition delayed the decision times. There was no significant effect of Task order (*b*=0.890, 95% CI=[−1.201, 2.981], *t* (44)=0.816, *p*=0.419). There was no significant interaction between Temperature condition and Task order (*b*=3.398, 95% CI=[−0.785, 7.581], *t* (44)=1.558, *p*=0.126).

### Discussion

In Experiment 3, we examined the cool temperature effect in two types of moral judgment tasks. However, we failed to obtain any clear evidence of an ambient temperature effect on moral judgment in either task, despite the fact that we obtained significant differences in the subjective ratings of the room warmth, body warmth, and level of comfort. Participants in the Cool temperature conditions felt their body was colder, thought the room was colder, and felt less comfort, but this did not affect their task performance. In this sense, we note that our findings in Experiment 3 provide a direct refutation of our own findings in Experiment 1, as well as a failure to conceptually replicate the notion of a cool temperature effect on moral judgment as obtained by Nakamura et al. (2014). Instead, the data add credence to the notion of limited reproducibility and/or generalizability of the phenomenon.

Notably, however, our ambient temperature manipulation in Experiment 3 was not strong enough to exert a physiological influence. Moreover, there was a significant interaction between task order and temperature condition. Though there were no clear tendencies to be observed from that interaction, it is possible that the exposure duration played a complex modulating role. It is possible that, for the cool temperature effect to obtain, it is not sufficient to induce a subjective (or psychological) sense of cold. In an effort to examine this point more closely, we conducted Experiment 4 in such a way as to strengthen the temperature manipulation to ensure a physiological influence.

## Experiment 4

Based on the discrepancy between the results of Experiment 1 and 3, we aimed to identify potential factors to explain the lack of a physiological influence from ambient temperature in Experiment 3. Notably, in Experiment 1, but not in Experiment 3, a two-step acclimation procedure was applied, raising the possibility that the acclimation is crucial to inducing the physiological effect. Thus, we decided in Experiment 4 to focus on the exposure duration to cold, comparing between short and long exposures to a cold environment. If the difference between Experiment 1 and Experiment 3 is due to the exposure duration, then the cool temperature should promote utilitarian judgment only in the long exposure condition. Also, though less likely to affect the results, we reverted back from a continuous rating scale (from −10 to +10) to a 7-point rating scale as the response dimension in the moral dilemma task.

### Method

#### Participants

Taking into consideration the minimal required samples sizes as determined in the previous experiments (effect size f of 0.309), we determined to collect at least 27 participants per experimental condition. In total, we collected data from 68 healthy Japanese undergraduate and graduate students (40 males and 28 females, age: *M_age_*=21.53, *SD_age_*=2.53). Participants were randomly assigned to one of the three different Temperature exposure conditions: No exposure to cool environment as Control (13 males, 3 females), 5min exposure as Short (14 males, 12 females), and 60min exposure as long (13 males, 13 females). The imbalance in the sample sizes was due to unexpected cancellation and constraints in running the experiments during the COVID-19 pandemic. The present study was conducted in February, March and April. All subjects were naïve to the purpose of the experiment. Each subject received compensation worth 1,500 yen for their participation. Written informed consent was obtained from all participants before the experiment.

#### Apparatus

A desktop computer with Psychopy software (version 3.1.2) controlled all events and data collection. All visual stimuli were presented on a 23-inch monitor, with a display resolution of 1920×1080 pixels.

### Measures

The measures were the same as in Experiment 1, except that the question of body warmth was added to the subjective measurements.

### Procedure

The flow of the procedure was similar as in Experiment 1. At the beginning of the experiment, the participants were given instructions about the experimental procedure in a waiting booth in the experiment room for about 10min to control their condition before the temperature manipulation. Informed consent was obtained from each participant during this period. The waiting booth temperature was set at 24°C (*M_temperature_*=23.55, *SD_temperature_*=0.74), the same temperature as the control condition in Experiment 1, through the room air conditioner with circulator and heater.

After the instruction, the skin temperature of the participant’s forehead was measured as the baseline (Time 1) of their physiological level. The experiment booth temperature was set to the standard for each experimental condition. Control condition was set at 24°C (*M_temperature_*=24.37, *SD_temperature_*=0.46), and short and long exposure conditions were set at 21°C (Short: *M_temperature_*=20.92, *SD_temperature_*=0.54; Long: (*M_temperature_*=20.84, *SD_temperature_*=0.56) through the room air conditioner with circulator and heater. The participants were exposed to the experimental temperature for a duration as according to the experimental condition. In the Control condition, the participants filled out the questionnaire about the subjective warmth and feelings of their comfort and arousal; also, their skin temperature was measured immediately as the manipulation check (Time 2). In the short exposure condition, participants waited for 5min alone in the experiment booth while relaxing to habituate to the environment. The manipulation check items were measured after 5min habituation period. In the long exposure condition, the participants also waited for 5min alone in the booth. After the short habituation, they performed two cognitive tasks unrelated to the moral dilemma task (a face memory task and a visual maze task) until they had stayed 60min in the experiment booth. The manipulation check items were measured after the two tasks were completed.

After the manipulation check, the participants in all three conditions performed the moral dilemma task. The participants judged the moral acceptability of the proposed utilitarian action in each dilemma using a 7-point scale (1 = Completely unacceptable; 7 = Completely acceptable), and their response times were measured. The ratings were made by mouse click. There was no time limit.

The skin temperature of the participants was measured again after the moral dilemma task as the final state (Time 3). With this, the experiment in the long exposure condition was completed. The experiments in the Control and short exposure condition were completed after the two cognitive tasks. The participants took about 90min to complete the entire set of experimental procedures. The study was conducted in accordance with the ethical principles of Kyushu University and approved by the Human Ethics Committee of the Faculty of Arts and Science (issue number 201902).

### Results

#### Manipulation Assessment

A one-way ANOVA was conducted on the subjective room warmth, body warmth, comfort, and arousal ratings after the habituation phase for each temperature exposure condition (Control vs. Short vs. Long) as the between-subject factor. Bonferroni’s multiple comparison was used for post-hoc analysis to the entire data set in all statistical analyses except when the assumption of equality of error variances was not met. Table 8 shows the means and 95% confidence intervals of room warmth, body warmth, and skin temperature at each time of manipulation check in the Control, short, and long exposure conditions. A summary of the results of the main effects of experimental temperature condition in the ANOVA of each manipulation check index is shown in Supplementary Table S4. The detailed descriptions of the statistical analyses with respect to the manipulation assessment of subjective indices are presented in the Supplementary Material. In brief, there was a significant main effect of temperature condition in room warmth, body warmth, and comfort rating.

**Table 8:** Means and 95% confidence intervals of room warmth rating, body warmth rating, comfort rating, arousal rating, and skin temperature in the Control, short exposure duration, and long exposure duration conditions in Experiment 4.

Measures		Control			Short			Long		
		Mean	95% CI		Mean	95% CI		Mean	95% CI	
Warmth	Room	4.188	3.899	4.476	3.231	2.968	3.493	2.615	2.291	2.939
	Body	4.563	4.177	4.948	3.692	3.355	4.030	3.077	2.736	3.418
Comfort		5.125	4.487	5.763	5.269	4.682	5.857	3.846	3.353	4.339
Arousal		4.000	3.488	4.512	4.115	3.732	4.499	3.923	3.402	4.445
Skin temp (Celsius)	Time 1	36.625	36.492	36.758	36.758	36.658	36.857	36.769	36.667	36.871
	Time 2	36.638	36.514	36.761	36.581	36.494	36.668	36.546	36.470	36.622
	Time 3	36.800	36.709	36.891	36.604	36.514	36.694	36.592	36.517	36.668

**Figure 5:**
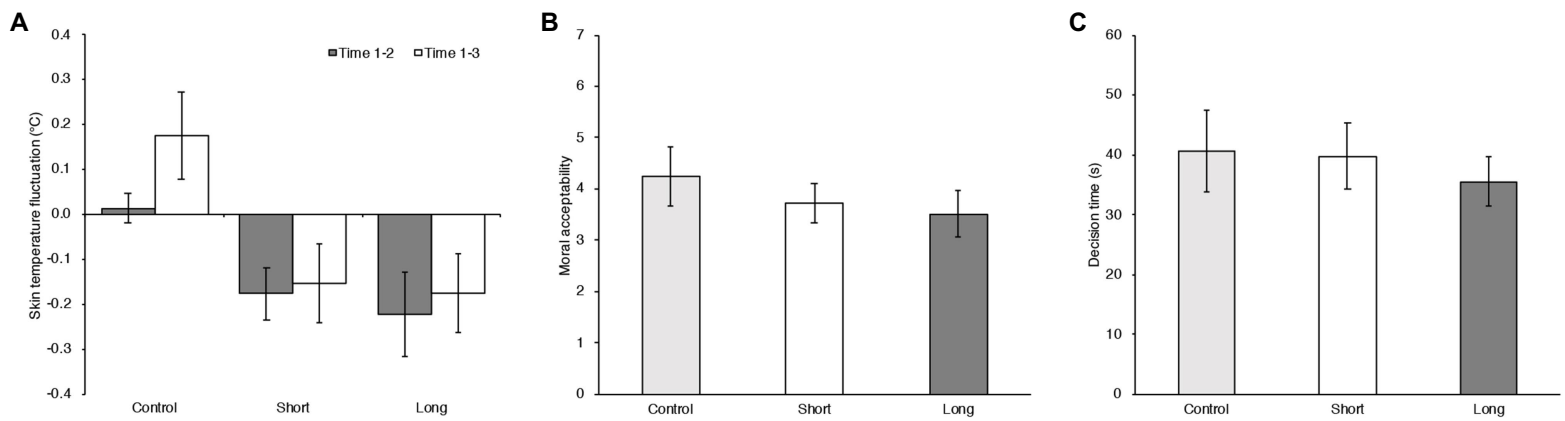
**(A)** Averaged differences in skin temperature between baseline and before the moral dilemma task (Time 1–2; gray bars) and between baseline and after the moral task (Time 1–3; white bars) in the Control, the short exposure duration, and the long exposure duration conditions in Experiment 4. Error bars indicate the 95% confidence intervals of the mean in each condition. **(B)** Mean moral acceptability ratings in the Control, short exposure duration, and long exposure duration conditions in Experiment 4. Error bars indicate the 95% confidence intervals of the mean in each condition. **(C)** Mean decision times for each condition in Experiment 4. Error bars indicate the 95% confidence intervals of the mean in each condition.

To assess the effect on physiological indices by temperature exposure duration, the amounts of change in skin temperature were calculated by subtracting each value after the experimental task phase (Time 3) and before the experimental task phase (Time 2) from that obtained in the baseline phase (Time 1). The indices of skin temperature fluctuation for each temperature exposure duration are shown in Figure 5A. Factorial RM ANOVA was computed on the skin temperature fluctuation with the within-subject factor measurement time [Before the experimental task (Time 1–2) vs. After the experimental task (Time 1–3)]). Temperature exposure duration was the between-subject factor. A significant main effect of temperature exposure duration was found, *F* (2, 65)=15.500, *MSE*=0.061, *p*<0.001, $\eta_{p}^{2}$=0.323, as well as a significant main effect of measurement time, *F* (1, 65)=16.381, *MSE*=0.012, *p*<0.001,$\eta_{p}^{2}$=0.201. There was significant effect of interaction between temperature exposure duration and measurement time, *F* (2, 65)=4.403, *MSE*=0.012, *p*=0.016, $\eta_{p}^{2}$=0.119. The results of simple main effect analysis showed that the effect of temperature exposure condition was significant in Time 1–2 (*F* (2, 65)=9.771, *MSE*=0.030, *p*<0.001,$\eta_{p}^{2}$=0.231) and Time 1–3 (*F* (2, 65)=16.413, *MSE*=0.043, *p*<0.001,$\eta_{p}^{2}$=0.336). Because Levene’s test of equality of error variances in Time 1–2 was significant (*F* (2, 65)=7.449, *p*=0.001), Games-Howell’s multiple comparison was used for post-hoc analysis in time 1–2. Post-hoc analysis in Time 1–2 revealed that participants in the long exposure condition (*M_LONG_*=−0.223, 95% CI=[−0.317, −0.129], *t* (30.367)=4.869, *adj. p*<0.001, Cohen’s *d*=1.919) and those in the short exposure condition (*M_SHORT_*=−0.177, 95% CI=[−0.234, −0.120], *t* (36.925)=5.936, *adj. p*<0.001, Cohen’s *d*=1.549) showed a decreased skin temperature as compared to participants in the Control condition (*M_CONTROL_*=0.013, 95% CI=[−0.020, 0.045]). There was no significant difference between participants in the long exposure condition and those in the short exposure condition (*t* (41.289)=0.860, *adj. p*=0.668, Cohen’s *d*=0.511). *Post hoc* analysis in Time 1–3 revealed that participants in the long exposure condition (*M_LONG_*=−0.177, 95% CI=[−0.265, −0.089], *t* (65)=5.317, *adj. p*<0.001, Cohen’s *d*=2.371) and those in the short exposure condition (*M_SHORT_*=−0.154, 95% CI=[−0.240, −0.068], *t* (65)=4.968, *adj. p*<0.001, Cohen’s *d*=2.224) had a decreased skin temperature as compared to participants in the Control condition (*M_CONTROL_*=0.175, 95% CI=[0.079, 0.271]). There was no significant difference between participants in the long exposure condition and those in the short exposure condition [*t* (65)=0.399, *adj. p*=1.000, Cohen’s *d*=0.155]. The effect of Measurement time was also significant for the participants in the Control condition, *F* (1, 65)=17.970, *MSE*=0.012, *p*<0.001,$\eta_{p}^{2}$=0.217. Compared with the mean of skin temperature fluctuation for Time 1–2 (*M_TIME1-2_*=0.013, 95% CI=[−0.020, 0.045]), the skin temperature for Time 1–3 was increased (*M_TIME1-3_*=0.175, 95% CI=[0.079, 0.271]).

Overall, the subjective reports about perception of coldness showed statistically significant increases, indicating that the perception became stronger with exposure time. Participants in the long exposure condition felt less comfort than the participants in the other conditions. Skin temperature fluctuation also proved that the temperature manipulations induced significant physiological effects. Taken together, the manipulation checks showed similar results as compared to the manipulation checks in Experiment 1.

#### Moral Judgment

The means and 95% confidence intervals of the moral judgment ratings and decision times in the Control, short, and long exposure duration conditions are shown in Figures 5B,C. A generalized linear mixed effect model with Poisson distribution was computed. Experimental condition, each scenario factor in dilemma task (Personal Force, Benefit Recipient, Evitability), comfort rating and the interaction between experimental condition and scenario factors up to three-way were modeled as fixed effects. The participants and stimuli (dilemma scenarios) were modeled as random effects. Categorical variables, Personal Force (Personal harm/Impersonal harm), Benefit recipient (Other-Beneficial/Self-Beneficial), and Evitability (Avoidable harm/Inevitable harm) were coded as −0.5/+0.5 contrasts, same as Experiment 1. Experimental condition was coded as a combination of two categorical variables C1 and C2 with −0.5/+0.5 contrasts. The Control condition was coded with C1: −0.5 and C2: −0.5; the short exposure duration condition with C1: +0.5 and C2: −0.5; and the long exposure duration condition with C1: −0.5 and C2: +0.5.

Variance of the random effects, regression coefficients (*b)* and 95% confidence intervals of the fixed effects, *z*-values and *p*-values are shown in Table 9. The main effect of the long exposure duration condition (C2) was significant (*b*=−0.198, 95% CI=[−0.395, 0.001]). The long duration of cool temperature exposure decreased utilitarian judgment more than did the other two conditions. Conversely, there was no significant effect of short exposure duration condition (C1: *b*=−0.163, 95% CI=[−0.350, 0.024]). There was a significant interaction between short exposure duration and the scenario factor of Evitability (C1: *b*=0.171, 95% CI=[0.009, 0.333]); however, the interaction between with the long exposure condition was not significant (C2: *b*=−0.070, 95% CI=[−0.234, 0.092]). As for the results of simple slope analysis by temperature condition, the Short exposure condition showed a significant effect in the Avoidable case (*b*=−0.248, 95% CI=[−0.458, −0.038]). The short duration of cool temperature exposure decreased utilitarian judgment when the dilemma situation was such that having a victim was avoidable. Conversely, the effect of the short exposure condition was not significant in the Inevitable case (*b*=−0.077, 95% CI=[−0.273, 0.119]). There was no significant interaction between temperature condition and any of the other scenario factors: Personal Force and temperature condition (C1: *b*=0.054, 95% CI=[−0.106, 0.213]; C2: *b*=0.060, 95% CI=[−0.100, 0.219]); Benefit Recipient and temperature condition (C1: *b*=0.056, 95% CI=[−0.106, 0.213]; C2: *b*=−0.102, 95% CI=[−0.265, 0.059]). Comfort rating also had no effect (*b*=0.000, 95% CI=[−0.055, 0.055]).

**Table 9:** Summary of the results from the generalized linear mixed effect model in Experiment 4.

Effect	Variance	*b*	95% CI		*z*	Value of *p*
**Fixed effects**						
Intercept		1.202^***^	0.928	1.475	8.755	< 0.001
Short exposure (C1)		−0.163	−0.350	0.024	−1.733	0.083
× Personal force		0.054	−0.106	0.213	0.664	0.507
× Benefit recipient		0.056	−0.106	0.218	0.681	0.496
× Evitability		0.171^*^	0.009	0.333	2.079	0.038
Long exposure (C2)		−0.198^*^	−0.395	0.001	−1.975	0.048
× Personal force		0.060	−0.100	0.219	0.738	0.460
× Benefit recipient		−0.102	−0.265	0.059	−1.248	0.212
× Evitability		−0.070	−0.234	0.092	−0.849	0.396
Personal force		0.139	−0.011	0.289	1.913	0.056
Benefit recipient		0.096	−0.054	0.247	1.317	0.188
Evitability		0.396^***^	0.245	0.547	5.436	< 0.001
Comfort		0.000	−0.055	0.055	0.001	0.999
**Random effects**						
Participants						
Intercept	0.070					
Stimuli						
Intercept	0.015					

* *=p<0.05*;

**=p<0.01;

*** *=p<0.001*.

*b = regression coefficients (not standardized); the range of the outcome variable was from 1 to 7*.

To confirm the temperature effect on the decision-making process, a generalized linear mixed effect model with Gaussian distribution was computed using the decision times. The experimental conditions (C1, C2) were modeled as fixed effects. The participants and stimuli were modeled as random effects. The result showed no significant effect, neither for the short exposure duration condition (C1: *b*=−0.848, 95% CI=[−8.458, 6.762], *t* (69.994)=−0.217, *p*=0.829), nor for the long exposure duration condition (C2: *b*=−5.083, 95% CI=[−12.693, 2.527], *t* (69.994)=−1.303, *p*=0.197).

### Discussion

Experiment 4 was conducted as a close replication of Experiment 1. The temperature manipulation proved to be successful, both in terms of subjective perceptions of coldness and feeling of comfort, and in terms of physiological effects as measured by skin temperature fluctuation. A cool temperature effect on moral judgment in moral dilemma task was observed when exposure duration was long. The short time duration interacted with the scenario factor of Evitability. Importantly, however, these effects in Experiment 4 show *less* utilitarian judgments as compared to the Control conditions, completely in opposition to Experiment 1. Thus, we failed to replicate the effect of temperature on the judgments in the moral dilemma task. The data indicated that the cool temperature effect on moral judgment is difficult to reproduce, to the point that it seems fair to question the robustness of the effect.

### Meta-Analysis of Experiments 1–4

The temperature effect as examined in the present series of experiments was not consistent. To assess the relative strength of the evidence overall, we conducted a Bayesian independent-samples *t* test, comparing the Control condition against the Experimental condition with the strongest manipulation in each experiment. For this purpose, we normalized the data by using standard or *z-*scores for each participant, using the population mean and population standard deviation of the respective experiment.

For the Experimental condition, we used data from the Cool condition in Experiment 1, the 3-part condition in Experiment 2, the Cool condition (of the moral dilemma task only) in Experiment 3, and the Long exposure duration condition in Experiment 4. The corresponding Control conditions in the four experiments served as the comparison for the independent-samples *t* test. The Bayesian testing was conducted following the guidelines and using the JASP software package provided by Wagenmakers et al. (2018a, 2018b).

**Figure 6:**
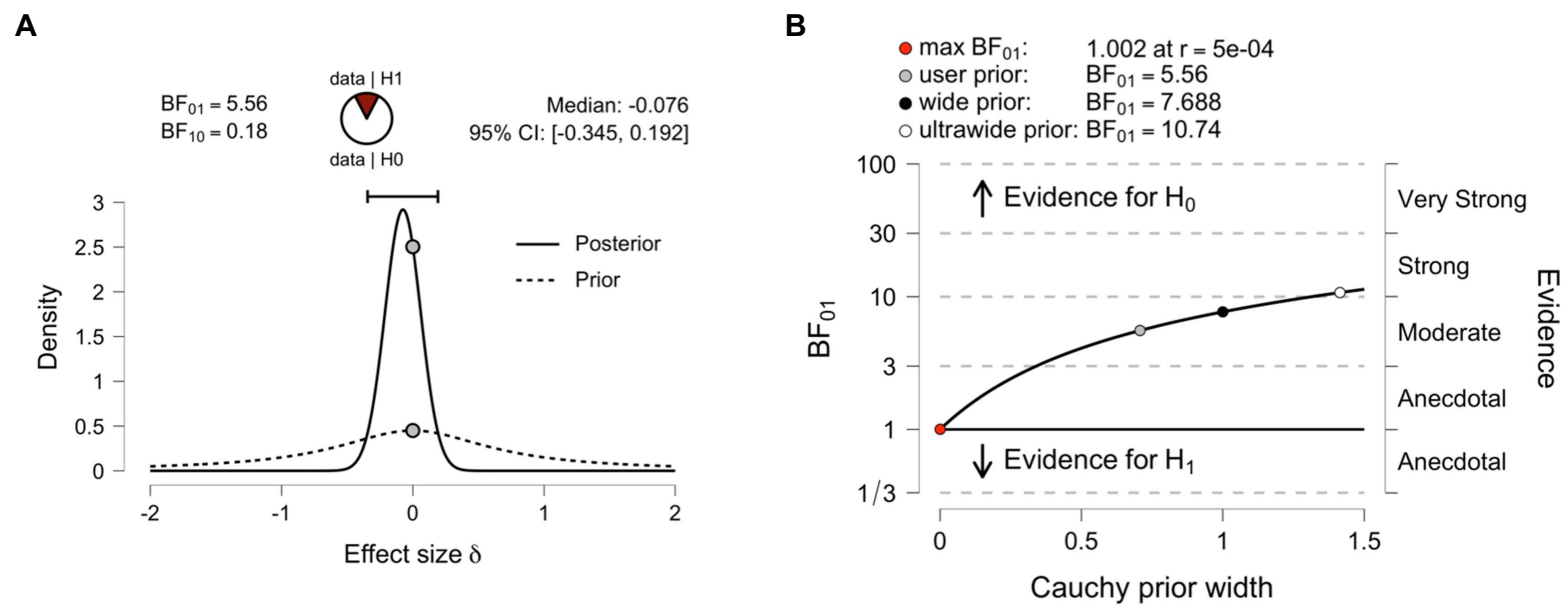
Meta-analysis of Experiments 1–4. The panels show inferential plots of a Bayesian independent-samples *t*-test, for normalized data (using *z*-scores) combined from Experiments 1–4 with control condition vs. (cool temperature) experimental condition. The left panel **(A)** shows the posterior and prior; the right panel **(B)** shows the Bayes factor robustness check.

Figure 6 shows the posterior and prior (left panel) and the Bayes factor robustness check (right panel) of the Bayesian independent-samples *t*-test, comparing the Control data vs. Experimental data from the four experiments in the present study. With a Bayes factor BF_01_ of 10.74 using an ultrawide prior, overall, the data tended to provide strong support in favor of the null hypothesis. Participants in the Control conditions (*M_CONTROL_*=−0.043, 95% CI=[−0.252, 0.167]) did not give different acceptability ratings in the moral dilemma task as compared to participants in the Experimental conditions (*M_EXPERIMENTAL_*=0.038, 95% CI=[−0.148, 0.224]).

## General Discussion

The present study examined in detail the effect of ambient and haptic temperature on social judgment, focusing on the effect of cold temperature in a moral dilemma task, following on from earlier work by Nakamura et al. (2014). In one of the four experiments here, we found a cool temperature that promoted utilitarian judgment, similar to the previous study. The remaining experiments, however, produced weak effects in the opposite direction or no effect of temperature on moral judgment. This occurred despite the fact that our temperature manipulations elicited reliable differences in perceptions of coldness, feelings of comfort, and physiological measurements of skin temperature.

A meta-analysis of the normalized data from all experiments, using Bayesian testing, provided firm evidence in favor of the null hypothesis. Taken together, our findings trace the limited reproducibility of effects from temperature on moral judgment and thus serve to caution against overinterpretation when psychologizing about the embodied “cold-heartedness” or “cool-headedness.”

One important caveat here is that we worked within a safe range of temperatures, between 21°C and 27°C, in line with the ethical guidelines at the universities where the experiments were carried out. In this setting, we followed temperature studies of social judgments that set cold temperature in the range of approximately between 20°C and 22°C (e.g., Gockel et al., 2014; Wang, 2017). However, the 21°C here reflects a cool temperature within the range used in this study, and could be interpreted as a relatively warm temperature in terms of general temperature. While this range allowed us to effectively elicit both psychological and physiological responses to the temperature conditions, it might not be strong enough to turn temperature into a salient stressor or trigger that could induce an effect on moral judgment. Thus, our findings suggest that the onset of psychological and physiological signatures of temperature does not co-occur with influences on moral judgment. Awareness of cold does not lead to a change in moral judgment. However, it is still possible that influences in the moral dilemma task arise outside the range of 21°C and 27°C, when temperature works as a more salient stressor. Especially, temperatures of less than 21°C should be examined to inspect the relationship between more salient cold temperature and moral judgment.

Hancock et al. (2007) suggested an inverse U-shaped relationship between the effect size and temperature intensity. The effects would be relatively weak in the comfort zone and rapidly become stronger outside this zone. Yeganeh et al. (2018) indicated that the direction of the effect becomes more stable and stronger as the temperature difference increases. From this perspective, the question remains open how an extreme cold temperature would affect performance in the moral dilemma task.

As a limitation of the present experimental procedures, we note that we conducted the manipulation checks several times in each experiment. Moreover, the participants were informed during the initial briefing toward obtaining informed consent that the study related to temperature. One interpretation of the present lack of effects from temperature, then, could be that our participants were on their guard and therefore less susceptible to any effects from temperature on moral judgment. Future studies should consider using deception, as employed by Nakamura et al. (2014), in order to examine how the awareness of temperature may modulate any effect on moral judgment.

The process of moral judgment in moral dilemma situations is explained from dual-process theory (Greene, 2007; Greene, 2009). In this theory, the decision in dilemma could be predicted according to whether automatic emotion or cognitive control predominates. Studies of moral dilemma revealed that manipulations that induce negative emotions like stress lead to the dominance of automatic emotion processing, and this would lead to suppressing utilitarian judgment (Starcke et al., 2012; Youssef et al., 2012). In our study, the cool conditions consistently elicited unpleasant emotions. Nevertheless, to the extent one might discern an effect of cool temperature in certain conditions (our Experiment 1 and the work by Nakamura et al., 2014), the tendency would be for cold to promote utilitarian judgment.

On the other hand, it should be noted that the moral dilemma task involves just one type of moral judgment and arguably a rather unusual case of decision-making in which participants are faced with a choice of life or death for multiple people. In particular, the option to save more people by sacrificing one victim in the moral dilemma task is called utilitarian judgment; however, this does not accurately reflect utilitarian thought in the strict sense. Specifically, it was pointed out that the “the greater good” aspect of the genuine idea of utilitarianism may not be reflected in the tendency to answer utilitarian judgments in the moral dilemma task (Kahane et al., 2015; Crone and Laham, 2017). Two separable dimensions have been identified regarding utilitarian thought in moral psychology (Kahane et al., 2018). One dimension reflects the essence of utilitarianism with impartial concern for “the greater good,” and the other dimension involves permissiveness toward instrumental harm. Strictly speaking, the moral judgments measured in this study may not have reflected a utilitarian tendency, but the acceptability of actively sacrificing victims to save others.

## Conclusion

In conclusion, for the present study, we note that it is an important finding for human society that moral judgment is not easily changed in a mild-range temperature environment. One direction for future research will be to investigate how temperature as a salient stressor impacts on decision-making in a variety of tasks involving moral judgment and social cognition.

## Data Availability Statement

The original contributions presented in the study are included in the article/Supplementary Material, further inquiries can be directed to the corresponding author.

## Ethics Statement

The studies involving human participants were reviewed and approved by the Ethics Committee of Senshu University (Issue No. 16-S001-2), the Ethics Committee of Hiroshima Shudo University (Issue No. 2017–10, and 2018–0003), and the Human Ethics Committee of the Faculty of Arts and Science, Kyushu University (Issue No. 201902). The patients/participants provided their written informed consent to participate in this study.

## Author Contributions

RS and MU conducted the data collection for Experiment 1. RS conducted the data collection for Experiments 2, 3 and 4, analyzed all the data, and prepared all the figures and tables. RS and JL wrote the manuscript. All authors contributed to the design of the study and reviewed and approved the manuscript.

## Funding

This work was supported by Grant-in-Aid for JSPS Fellows (18J13558) to RS from the Japan Society for the Promotion of Science, Japan, and JSPS KAKENHI grant number JP15K21704 to SFN.

## Conflict of Interest

The authors declare that the research was conducted in the absence of any commercial or financial relationships that could be construed as a potential conflict of interest.

## Publisher’s Note

All claims expressed in this article are solely those of the authors and do not necessarily represent those of their affiliated organizations, or those of the publisher, the editors and the reviewers. Any product that may be evaluated in this article, or claim that may be made by its manufacturer, is not guaranteed or endorsed by the publisher.

## Supplementary Material

Datasheet: Complete data set for manipulation check and moral judgment data in the present study, organized in five Data Sheets: Experiment 1; Experiment 2; Experiment 3; Experiment 4; and Meta-analysis. Original stimuli IDs used in the moral dilemma task and moral image task were also included in the datasheet.

Supplementary Results: Manipulation assessment of subjective indices in Experiments 1 to 4, with Supplementary Tables S1–S4.
