# Supplementary material for: The Role of Temperature in Moral Decision-Making: Limited Reproducibility
Source: Front Psychol. 2021 Sep 28;12:681527. doi: 10.3389/fpsyg.2021.681527 (PMC8506165; doi:10.3389/fpsyg.2021.681527)
Supplement: Supplementary file 2 — Supplementary Results: Manipulation assessment of subjective indices in Experiments 1 to 4, with Supplementary Tables S1–S4. [file Table_1.DOCX]

Supplementary Results:

**Manipulation Assessment of Subjective Indices**

**EXPERIMENT 1 RESULTS – manipulation assessment of subjective indices**

A two-way analysis of variance (ANOVA) was conducted on the subjective room warmth, comfort, and arousal rating after the habituation phase for each temperature condition (Cool vs. Control vs. Hot) and season (Summer vs. Winter) as Between-Subjects factors. Bonferroni’s multiple comparison was used for post-hoc analysis to the entire data set in all statistical analyses except when the requirement of equal error variances was not met. **Table 1** shows the means and 95% confidence intervals of room warmth, comfort, and arousal rating, and skin temperature at each time of manipulation check in the Cool, Control, and Hot room temperature conditions and the Summer and Winter seasons. A summary of the results of the main effects of experimental temperature condition in ANOVA of each manipulation check index is shown in **Table S1**.

In the room warmth ratings, a significant main effect of temperature condition was found, *F*(2, 73) = 18.660, *MSE* = 1.430, *p* < .001,η_p_^2^ = .338, as well as a significant main effect of season, *F*(1, 73) = 17.131, *MSE* = 1.430, *p* < .001,η_p_^2^ = .190. There was no significant effect of the interaction between temperature condition and season, *F*(2, 73) = 0.440, *MSE* = 1.430, *p* = .646,η_p_^2^ = .012. Post-hoc analysis revealed that Hot temperature participants felt warmer (*M_HOT_* = 4.556, 95% CI = [4.027, 5.085]) than Control temperature participants (*M_CONTROL_* = 3.333, 95% CI = [2.820, 3.847], *t*(76) = 3.422, *adj.p* = .003, Cohen’s *d* = 0.917) and Cool temperature participants (*M_COOL_* = 2.520, 95% CI = [1.987, 3.053], *t*(76) = 5.588, *adj.p* < .001, Cohen’s *d* = 1.528). There was no significant difference between Cool temperature participants and Control temperature participants (*t*(76) = 2.233, *adj.p* = .086, Cohen’s *d* = 0.610). Compared with the mean of room warmth feeling for Summer participants (*M_SUMMER_* = 2.914, 95% CI = [2.512, 3.317]), the room warmth for Winter participants was warmer (*M_WINTER_* = 3.955, 95% CI = [3.451, 4.458]).

In the comfort ratings, a significant main effect of temperature condition was found, *F*(2, 73) = 14.514, *MSE* = 2.184, *p* < .001,η_p_^2^ = .285, as well as a significant effect of the interaction between temperature condition and season, *F*(2, 73) = 3.277, *MSE* = 2.184, *p* = .043,η_p_^2^ = .082. There was no significant main effect of season, *F*(1, 73) = 0.711, *MSE* = 2.184, *p* = .402,η_p_^2^ = .010. As for the results of simple main effect analysis, the effect of temperature condition was significant in Summer season participants, *F*(2, 73) = 12.788, *MSE* = 2.184, *p* < .001,η_p_^2^ = .259. Post-hoc analysis revealed that Cool temperature participants felt less comfort (*M_COOL_* = 2.333, 95% CI = [1.267, 3.400] ) than Control temperature participants (*M_CONTROL_* = 4.857, 95% CI = [3.907, 5.807], *t*(73) = 3.997, *adj.p* < .001, Cohen’s *d* = 2.323) and Hot temperature participants (*M_HOT_* = 5.500, 95% CI = [4.673, 6.327], *t*(73) = 4.859, *adj.p* < .001, Cohen’s *d* = 2.107). There was no significant difference between Hot temperature participants and Control temperature participants (*t*(73) = 1.106, *adj.p* = .817, Cohen’s *d* = 0.426). The effect of season was also significant in the Cool temperature condition, *F*(1, 73) = 6.268, *MSE* = 2.184, *p* = .015,η_p_^2^ = 079. Compared with the mean of comfort feeling for Winter participants (*M_WINTER_* = 3.875, 95% CI = [3.057, 4.693]), the Summer participants felt more uncomfortable (*M_SUMMER_* = 2.333, 95% CI = [1.267, 3.400]).

In the arousal ratings, there was no significant effect of the temperature condition, *F*(2, 73) = 1.347, *MSE* = 2.243, *p* = .266,η_p_^2^ = .036, nor of the season, *F*(1, 73) = 3.488, *MSE* = 2.243, *p* = .066,η_p_^2^ = .046. There was also no interaction between temperature condition and season, *F*(2, 73) = 0.189, *MSE* = 2.243, *p* = .829, η_p_^2^ = .005.

**EXPERIMENT 2 RESULTS – manipulation assessment of subjective indices**

A one-way ANOVA was conducted on the subjective room warmth, body warmth, forehead warmth, neck warmth, hand warmth, comfort, and arousal rating after the habituation phase for each manipulation condition (Control vs. 1 part vs. 3 parts) as a Between-Subjects factor. Bonferroni’s multiple comparison was used for post-hoc analysis to the entire data set in all statistical analyses except when the assumption of equality of error variances was violated. **Table 3** shows the means and 95% confidence intervals of room warmth, body warmth, forehead warmth, neck warmth, hand warmth, comfort, and arousal rating, and skin temperature at each time of manipulation check among the Control, 1 part, and 3 parts manipulation conditions. A summary of the results of the main effects of the experimental temperature condition in the ANOVA of each manipulation check index is shown in **Table S2**.

In the room warmth ratings, there was no significant main effect of manipulation condition, *F*(2, 79) = 0.728, *MSE* = 0.839, p = .486,η_p_^2^ = .018. In the body warmth rating, a significant main effect of manipulation condition was found, *F*(2, 79) = 14.523, *MSE* = 0.903, *p* < .001,η_p_^2^ = .269. Post-hoc analysis revealed that participants in the 3 parts condition felt colder (*M_3 PARTS_* = 2.407, 95% CI = [2.093, 2.722]) than participants in the 1 part condition (*M_1 PART_* = 3.185, 95% CI = [2.821, 3.549], t(79) = 3.008, *adj.p* < .011, Cohen’s *d* = 0.807) and participants in the Control condition (*M_CONTROL_* = 3.786, 95% CI = [3.360, 4.212], *t*(79) = 5.379, *adj.p* < .001, Cohen’s *d* = 1.430). There was no significant difference between participants in the 1 part condition and those in the Control condition (*t*(79) = 2.343, *adj.p* = .065, Cohen’s *d* = 0.623).

In the forehead warmth ratings, a significant main effect of manipulation condition was found, *F*(2, 79) = 49.771, *MSE* = 0.720, *p* < .001,η_p_^2^ = .558. Post-hoc analysis revealed that participants in the 3 parts condition felt colder (*M_3 PARTS_* = 1.963, 95% CI = [1.707, 2.219) than participants in the 1 part condition (*M_1 PART_* = 4.111, 95% CI = [3.777, 4.446], *t*(79) = 9.302, *adj.p* < .001, Cohen’s *d* = 2.496) and participants in the Control condition (*M_CONTROL_* = 3.750, 95% CI = [3.361, 4.139], *t*(79) = 7.808, *adj.p* < .001, Cohen’s d = 2.076). There was no significant difference between participants in the 1 part condition and those in the Control condition (*t*(79) = 1.578, *adj.p* = .356, Cohen’s *d* = 0.420).

In the neck warmth ratings, a significant main effect of manipulation condition was found, *F*(2, 79) = 26.639, *MSE* = 0.940, *p* < .001,η_p_^2^ = .403. Post-hoc analysis revealed that participants in the 3 parts condition (*M_3 PARTS_* = 2.370, 95% CI = [2.021, 2.719], *t*(79) = 6.230, *adj.p* < .001, Cohen’s *d* = 1.657) and participants in the 1 part condition (M*_1 PART_* = 2.333, 95% CI = [1.909, 2.757], *t*(79) = 6.372, *adj.p* < .001, Cohen’s *d* = 1.694) felt colder than participants in the Control condition (*M_CONTROL_* = 4.000, 95% CI = [3.635, 4.365]). There was no significant difference between participants in the 3 parts condition and those in the 1 part condition (*t*(79) = 0.140, *adj.p* = 1.000, Cohen’s *d* = 0.038).

In the hand warmth ratings, a significant main effect of manipulation area condition was found, (*F*(2, 79) = 60.073, *MSE* = 0.702, *p* < .001,η_p_^2^ = .603. Post-hoc analysis revealed that participants in the 3 parts condition felt colder (*M_3 PARTS_* = 1.889, 95% CI = [1.573, 2.205]) than participants in the 1 part condition (M*_1 PART_* = 4.222, 95% CI = [3.926, 4.519], *t*(79) = 10.234, *adj.p* < .001, Cohen’s *d* = 2.746) and those in the Control condition (*M_CONTROL_* = 3.821, 95% CI = [3.456, 4.187], *t*(79) = 8.553, *adj.p* < .001, Cohen’s *d* = 2.274). There was no significant difference between participants in the 1 part condition and those in the Control condition (*t*(79) = 1.774, *adj.p* = .240, Cohen’s *d* = 0.472).

In the comfort ratings, a significant main effect of manipulation area condition was found, *F*(2, 79) = 3.768, *MSE* = 1.483, *p* = .027,η_p_^2^ = .087. Post-hoc analysis revealed that participants in the 3 parts condition felt less comfort (*M_3 PARTS_* = 3.963, 95% CI = [3.505, 4.421]) than those in the Control condition (*M_CONTROL_* = 4.857, 95% CI = [4.366, 5.348], *t*(79) = 2.723, *adj.p* = .024, Cohen’s *d* = 0.724). There was no significant difference between participants in the 3 parts condition and those in the 1 part condition (*M_1 PART_* = 4.519, 95% CI = [4.037, 5.001], *t*(79) = 1.676, *adj.p* = .293, Cohen’s *d* = 0.450), nor between participants in the 1 part condition and those in the Control condition (*t*(79) = 1.031, *adj.p* = .917, Cohen’s *d* = 0.274).

In the arousal ratings, a significant main effect of manipulation condition was found, (*F*(2, 79) = 4.607, *MSE* = 1.255, *p* = .013,η_p_^2^ = .104. Post-hoc analysis revealed that participants in the 1 part condition felt less arousal (*M_1 PART_* =3.407, 95% CI = [3.009, 3.806]) than those in the 3 parts condition (*M_3 PARTS_* = 4.259, 95% CI = [3.827, 4.692], *t*(79) = 2.794, *adj.p* = .020, Cohen’s *d* = 0.750). There was no significant difference between participants in the 3 parts condition and those in the Control condition (*M_CONTRO_*_L_ = 4.143, 95% CI = [3.663, 4.622], *t*(79) = 0.385, *adj.p* = 1.000, Cohen’s *d* = 0.102), nor between participants in the 1 part condition and those in the Control condition (*t*(79) = 2.434, *adj.p* = .052, Cohen’s *d* = 0.647).

**EXPERIMENT 3 RESULTS – manipulation assessment of subjective indices**

Welch's independent t-test was computed on the subjective room warmth, body warmth, comfort, and arousal rating after the habituation phase for each temperature condition (Control vs. Cool) as the Between-Subjects factor. **Table 5** shows the means and 95% confidence intervals of room warmth, body warmth, comfort, and arousal rating, and skin temperature at each time of manipulation check in the Control and Cool temperature conditions. A summary of the results of the main effects of experimental temperature condition in t-tests of each manipulation check index is also shown in **Table S3**.

In the room warmth ratings, we found a significant difference between condition, *t*(44.517) = 5.019, *p* < .001, Cohen’s *d* = 1.425. Participants in the Cool condition felt colder (*M_COOL_* = 2.958, 95% CI = [2.642, 3.275]) than participants in the Control condition (*M_CONTROL_* = 3.958, 95% CI = [3.695, 4.221]). Also in the body warmth ratings, we found a significant difference between conditions, *t*(45.877) = 4.263, *p* < .001, Cohen’s *d* = 1.210. Participants in the Cool condition felt more body coolness (*M_COOL_* = 3.333, 95% CI = [2.968, 3.699]) than participants in the Control condition (*M_CONTROL_* = 4.375, 95% CI = [4.028, 4.722]).

In the comfort ratings, we found a significant difference between conditions, (41.266) = 2.584, *p* = .013, Cohen’s *d* = 0.734. Participants in the Cool condition felt less comfort (*M_COOL_* = 4.333, 95% CI = [3.680, 4.987]) than those in the Control condition (*M_CONTROL_* = 5.333, 95% CI = [4.874, 5.793]). In the arousal ratings, there was no significant difference between conditions, *t*(42.368) = 0.932, *p* = .357, Cohen’s *d* = 0.265.

**EXPERIMENT 4 RESULTS – manipulation assessment of subjective indices**

A one-way ANOVA was conducted on the subjective room warmth, body warmth, comfort, and arousal ratings after the habituation phase for each temperature exposure condition (Control vs. Short vs. Long) as the Between-Subjects factor. Bonferroni’s multiple comparison was used for post-hoc analysis to the entire data set in all statistical analyses except when the assumption of equality of error variances was not met. **Table 8** shows the means and 95% confidence intervals of room warmth, body warmth, and skin temperature at each time of manipulation check in the Control, Short, and Long exposure conditions. A summary of the results of the main effects of experimental temperature condition in the ANOVA of each manipulation check index is shown in **Table S4**.

In the room warmth ratings, a significant main effect of temperature exposure duration was found, *F*(2, 65) = 25.499, *MSE* = 0.480, *p* < .001,η_p_^2^ = .440. Because Levene’s Test of Equality of Error Variances in temperature condition was significant (*F*(2, 65) = 3.171, *p* = .049), Games-Howell’s multiple comparison was used for post-hoc analysis. Post-hoc analysis revealed that participants in the Long exposure condition (*M_LONG_* = 2.615, 95% CI = [2.291, 2.939]) felt colder than those in the Short exposure condition (*M_SHORT_* = 3.231, 95% CI = [2.968, 3.493], *t*(44.948) = 3.032, *adj.p* = .011, Cohen’s *d* = 0.875) and those in the Control condition (*M_CONTROL_* = 4.188, 95% CI = [3.899, 4.476], *t*(39.551) = 7.551, *adj.p* < .001, Cohen’s *d* = 2.235). Participants in the Short exposure condition felt colder than those in the Control condition, *t*(36.238) = 5.127, *adj.p* < .001, Cohen’s *d* = 1.360.

In the body warmth ratings, a significant main effect of temperature exposure duration was found, *F*(2, 65) = 16.428, *MSE* = 0.666, *p* < .001,η_p_^2^ = .336. Post-hoc analysis revealed that participants in the Long exposure condition (*M_LONG_* = 3.077, 95% CI = [2.736, 3.418]) felt more body coolness than those in the Short exposure condition (*M_SHORT_* = 3.692, 95% CI = [3.355, 4.030], *t*(65) = 2.718, *adj.p* = .025, Cohen’s *d* = 0.742) and those in the Control condition (*M_CONTROL_* = 4.563, 95% CI = [4.177, 4.948], t(65) = 5.727, adj.p < .001, Cohen’s d = 1.792). Participants in the Short exposure condition felt more body coolness than participants in the Control condition, *t*(65) = 3.355, *adj.p* = .004, Cohen’s *d* = 1.050.

In the comfort ratings, a significant main effect of temperature exposure duration was found, *F*(2, 65) = 8.763, *MSE* = 1.727, *p* < .001,η_p_^2^ = .212. Post-hoc analysis revealed that participants in the Long exposure condition (*M_LONG_* = 3.846, 95% CI = [3.353, 4.339]) felt less comfort than those in the Short exposure condition (*M_SHORT_* = 5.269, 95% CI = [4.682, 5.857], *t*(65) = 3.904, adj.p < .001, Cohen’s d = 1.067) and those in the Control condition (*M_CONTROL_* = 5.125, 95% CI = [4.487, 5.763], *t*(65) = 3.063, *adj.p* = .010, Cohen’s *d* = 0.958). There was no significant difference between participants in the Short exposure condition and those in the Control condition, (*t*(65) = 0.345, *adj.p* = 1.000, Cohen’s *d* = 0.108).

In the arousal ratings, there was no main effect of temperature exposure duration, *F*(2, 65) = 0.201, *MSE* = 1.208, *p* = .818,η_p_^2^ = .006.

Table S1. *F*-values and *p*-values, and summary of the multiple comparisons of main effects of the experimental temperature condition in the ANOVA for each subjective manipulation check index in Experiment 1.

|  |  |  |  |  |  |  |  |
| --- | --- | --- | --- | --- | --- | --- | --- |
| **Measures** | **Main effect (and Interaction)** |  | ***F*** |  | ***p*** |  | **Multiple comparison** |
|  |  |  |  |  |  |  |  |
|  |  |  |  |  |  |  |  |
| Room  warmth | Temperature |  | 18.660*** |  | < .001 |  | Cool***, Control** < Hot |
|  | × Season |  | 0.440 |  | .646 |  |  |
| Comfort | Temperature |  | 14.514*** |  | < .001 |  |  |
|  | × Season |  | 3.277* |  | .043 |  | Cool < Control***, Hot***  in Summer condition |
| Arousal | Temperature |  | 1.347 |  | .266 |  |  |
|  | × Season |  | 0.189 |  | .829 |  |  |

Note: * = *p* < .05; ** = *p* < .01; *** = *p* < .001

Table S2. *F*-values and *p*-values, and summary of the multiple comparisons of main effects of the experimental temperature condition in the ANOVA for each subjective manipulation check index in Experiment 2.

| **Measures** |  | ***F*** |  | ***p*** | **Multiple comparison** |
| --- | --- | --- | --- | --- | --- |
|  |  |  |  |  |  |
|  |  |  |  |  |  |
| Warmth | Room | 0.728 |  | .486 |  |
|  | Body | 14.523*** |  | <. 001 | 3 parts < 1 part*, Control*** |
|  | Forehead | 49.711*** |  | < .001 | 3 parts < 1 part***, Control*** |
|  | Neck | 26.639*** |  | < .001 | 3 parts***, 1 part*** < Control |
|  | Hand | 60.073*** |  | < .001 | 3 parts < 1 part***, Control*** |
| Comfort |  | 3.768* |  | .027 | 3 parts < Control* |
| Arousal |  | 4.607* |  | .013 | 1 parts < 3 parts* |

Note: * = *p* < .05; ** = *p* < .01; *** = *p* < .001

Table S3. *t*-values and *p*-values of the main effect of experimental temperature condition in the *t-*test for each manipulation check index in Experiment 3.

| **Measures** |  |  | ***t*** |  | ***p*** |  | **Comparison** |
| --- | --- | --- | --- | --- | --- | --- | --- |
|  |  |  |  |  |  |  |  |
|  |  |  |  |  |  |  |  |
| Warmth | Room |  | 5.019*** |  | < .001 |  | Cool < Control |
|  | Body |  | 4.263*** |  | < .001 |  | Cool < Control |
| Comfort |  |  | 2.584* |  | .013 |  | Cool < Control |
| Arousal |  |  | 0.932 |  | .357 |  |  |

Note: * = *p* < .05; ** = *p* < .01; *** = *p* < .001

Table S4. *F*-values and *p*-values, and summary of the multiple comparisons of main effects of the experimental temperature condition in the ANOVA for each subjective manipulation check index in Experiment 4.

| **Measures** |  |  | ***F*** |  | ***p*** |  | **Multiple comparison** |
| --- | --- | --- | --- | --- | --- | --- | --- |
|  |  |  |  |  |  |  |  |
|  |  |  |  |  |  |  |  |
| Warmth | Room |  | 25.499*** |  | < .001 |  | Long < Short*, Control*** Short < Control*** |
|  | Body |  | 16.428*** |  | < .001 |  | Long < Short*, Control*** Short < Control** |
| Comfort |  |  | 8.763*** |  | < .001 |  | Long < Short*, Control*** |
| Arousal |  |  | 0.201 |  | .818 |  |  |

Note: * = *p* < .05; ** = *p* < .01; *** = *p* < .001
